# Interferon‐γ inducible protein 30 promotes the epithelial–mesenchymal transition‐like phenotype and chemoresistance by activating EGFR/AKT/GSK3β/β‐catenin pathway in glioma

**DOI:** 10.1111/cns.14334

**Published:** 2023-07-05

**Authors:** Ying Chen, Hui Xu, Pei Yu, Qing Wang, Shenggang Li, Fufu Ji, Chunwang Wu, Qing Lan

**Affiliations:** ^1^ Department of Neurosurgery The Second Affiliated Hospital of Soochow University Suzhou Jiangsu P.R. China

**Keywords:** chemoresistance, epithelial–mesenchymal transition‐like, glioma, interferon‐γ inducible protein 30, temozolomide

## Abstract

**Aims:**

Previous studies have indicated that IFI30 plays a protective role in human cancers. However, its potential roles in regulating glioma development are not fully understood.

**Methods:**

Public datasets, immunohistochemistry, and western blotting (WB) were used to evaluate the expression of IFI30 in glioma. The potential functions and mechanisms of IFI30 were examined by public dataset analysis; quantitative real‐time PCR; WB; limiting dilution analysis; xenograft tumor assays; CCK‐8, colony formation, wound healing, and transwell assays; and immunofluorescence microscopy and flow cytometry.

**Results:**

IFI30 was significantly upregulated in glioma tissues and cell lines compared with corresponding controls, and the expression level of IFI30 was positively associated with tumor grade. Functionally, both in vivo and in vitro evidence showed that IFI30 regulated the migration and invasion of glioma cells. Mechanistically, we found that IFI30 dramatically promoted the epithelial–mesenchymal transition (EMT)‐like process by activating the EGFR/AKT/GSK3β/β‐catenin pathway. In addition, IFI30 regulated the chemoresistance of glioma cells to temozolomide directly via the expression of the transcription factor Slug, a key regulator of the EMT‐like process.

**Conclusion:**

The present study suggests that IFI30 is a regulator of the EMT‐like phenotype and acts not only as a prognostic marker but also as a potential therapeutic target for temozolomide‐resistant glioma.

## Graphical abstract

In the present study, we demonstrated that IFI30 contributes to the activation of the EGFR/AKT/GSK3β pathway by enhancing the nuclear translocation of β‐catenin. IFI30‐mediated EGFR/AKT/GSK3β/β‐catenin signaling not only promotes the epithelial–mesenchymal transition‐like phenotype by upregulating the expression of Slug, but also subsequently enhanced invasion and chemoresistance of glioma cells.

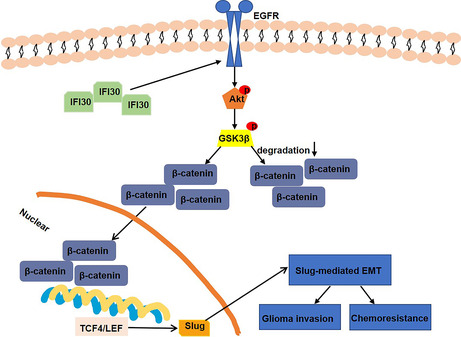

## 1 INTRODUCTION

Glioma accounts for approximately 81% of primary brain tumors^1^; it has a high incidence rate and a poor prognosis.^2^ The most prevalent subtype, glioblastoma (GBM), accounts for 57.7% of gliomas.^3^ Temozolomide (TMZ) is a first‐line chemotherapeutic agent for glioma patients and the only chemotherapeutic drug that has been confirmed to significantly prolong overall survival (OS),^4^ yet the median survival after receiving TMZ treatment combined with surgical resection, radiotherapy,^5^, ^6^ targeted therapy, and supportive care is only 14–16 months for GBM patients,^7^, ^8^, ^9^, ^10^ with a 5‐year recurrence rate as high as 90%.^11^ The reason for this low survival rate and high recurrence rate is largely due to its invasive behavior and resistance to current treatments, including TMZ,^7^ owing to its cellular and molecular heterogeneity.^12^ Therefore, there is an urgent need to investigate the key molecular mechanisms involved in the malignant progression of glioma to provide valuable prognostic biomarkers and therapeutic targets.

Genotyping and expression profiling analyses have demonstrated that GBM may be categorized into three subclasses (proneural/neural, classical, and mesenchymal) depending on the gene expression signatures.^13^ The proneural/neural subtype is associated with a favorable prognosis,^14^ while the mesenchymal subtype is highly invasive and has poor outcomes.^15^ Furthermore, nonmesenchymal subtypes of tumors typically acquire mesenchymal features at the time of recurrence.^11^ A shift toward the mesenchymal subtype appears to be a common pattern in disease progression, and this process is similar to epithelial–mesenchymal transition (EMT) in cancer cells, which facilitates the acquisition of more aggressive features.^16^ EMT has been identified as one of the mechanisms that confers cancer cell stemness, drug resistance, and immunosuppression during glioma development.^17^, ^18^ Although the central nervous system (CNS) lacks this critical tissue component,^19^ a range of changes in EMT‐associated activators, including decreased expression of various epithelial markers (occludins, E‐cadherin, ZO‐1) and increased expression of mesenchymal markers (N‐cadherin, vimentin, and fibronectin), were observed during glioma progression.^20^ Given these observations, the terms “proneural‐to‐mesenchymal transition” or the “EMT‐like” process have been proposed.^21^, ^22^ A better understanding of the molecular regulation of the EMT‐like process during tumor spreading will help to provide potential therapeutic interventions to target this program when treating glioma.

Whole‐exon and whole‐transcriptome sequencing of patients with metastatic tumors were used to demonstrate a strong correlation between the expression of interferon‐induced genes (ISGs) and EMT in a recent study. In addition, they determined that interferon‐induced tetratricopeptide repeat 5 (IFIT5) regulated by IFN‐γ is involved in the degradation of unique miRNAs that can promote EMT, leading to prostate cancer metastasis.^23^ There are more than 200 ISGs that play different roles in tumor promotion or antitumor activity in different diseases.^24^ A recent study carried out an analysis on glioma patients from the Chinese Glioma Genome Atlas (CGGA) and The Cancer Genome Atlas (TCGA) cohorts and identified IFI30 as a promising prognostic gene in glioma among ISGs.^25^

IFI30 encodes gamma‐interferon‐inducible lysosomal thiol reductase, which is the only enzyme known to catalyze disulfide bond reduction in the endocytic pathway.^26^, ^27^ The most well‐known function of IFI30 is the enhancement of MHC class II‐restricted antigen processing, which is essential for the activation of CD4^+^ T lymphocytes.^28^ In addition, IFI30 plays an important role in antibacterial infection, production of reactive oxygen species, induction of autophagy, and inhibition of the entry of selected enveloped RNA viruses.^29^ In addition, recent studies have revealed the aberrant expression of IFI30 in several types of cancers, such as diffuse large B‐cell lymphoma (DLBCL), melanoma, and breast cancer.^30^ However, the role of IFI30 in human glioma is still poorly understood.

In this study, the effect of IFI30 on glioma was investigated. Analysis of public databases and glioma tissues showed that IFI30 was an independent predictor for glioma, as high IFI30 mRNA expression was associated with poor patient outcomes. Furthermore, we demonstrated that IFI30 promoted the proliferation and migration of glioma cells. In addition, IFI30 promoted the EMT‐like process in gliomas via the EGFR/AKT/GSK3β/β‐catenin signaling pathway both in vitro and in vivo. Finally, we showed that IFI30 could be upregulated by TMZ and that the EMT‐like process was involved in TMZ resistance. The current study suggests that IFI30 could be a potential therapeutic target for glioma.

## 2 MATERIALS AND METHODS

Details are clarified in the Supplementary Materials and Methods (Data S1).

## 3 RESULTS

### 3.1 IFI30 is overexpressed and associated with a poor prognosis in gliomas

**FIGURE 1:**
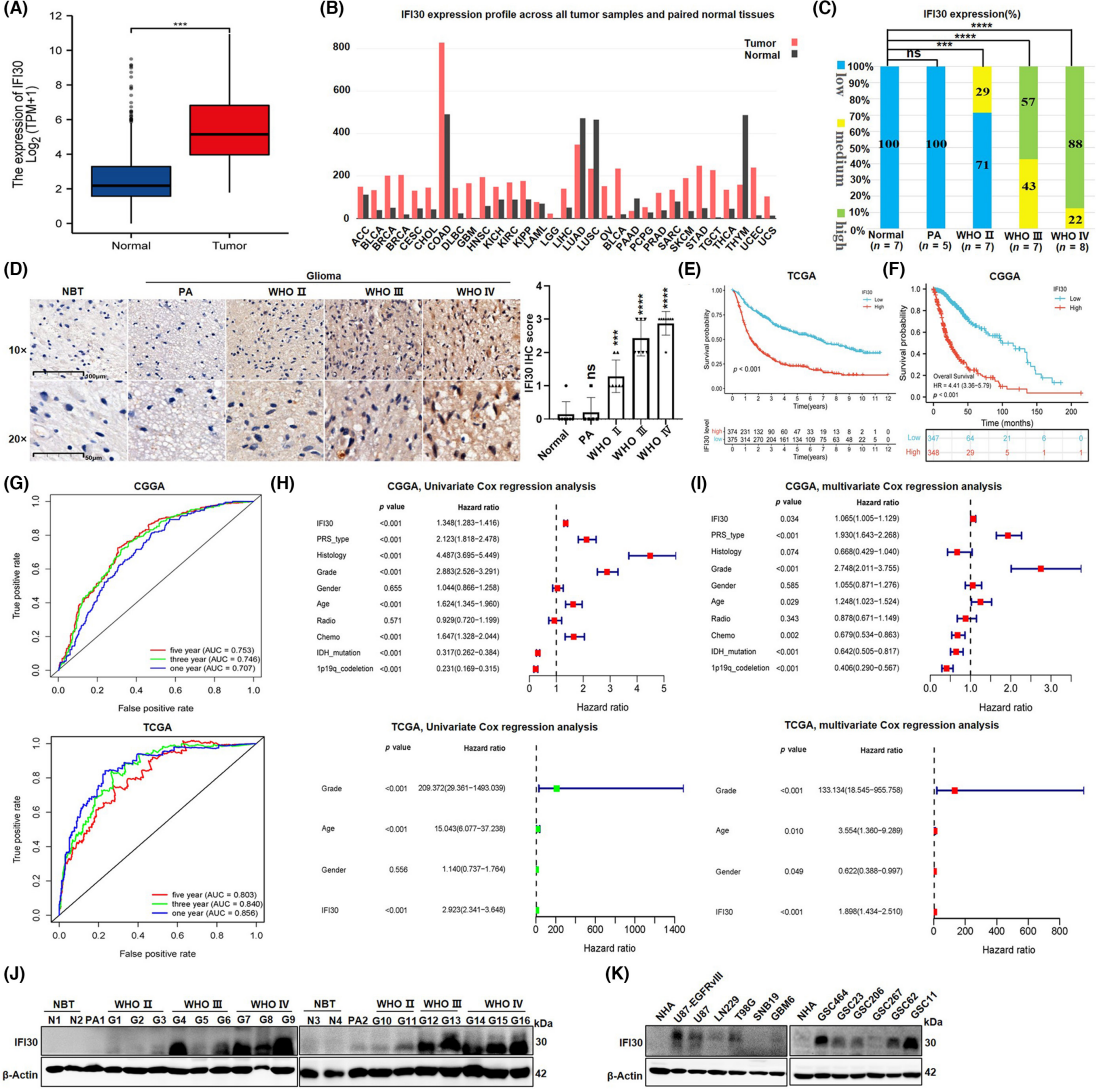
IFI30 is overexpressed and associated with a poor prognosis in gliomas. (A) The expression level of IFI30 mRNA was increased in glioma tissues compared with NBTs. IFI30 mRNA expression data were derived from the RNA sequencing array of the TCGA and GTEx databases. (B) IFI30 expression was analyzed in GBM, LGG, and corresponding paracancerous tissues with the GEPIA webserver. (C) Semiquantitative analysis of the IHC results of IFI30. (D) Representative IHC images of IFI30 protein in glioma tissues with different histological grades, PA tissues, and NBTs. Bar = 100 μm, and quantification of the intensity of staining was performed with ImageJ software. OS curves of IDH wild‐type GBM patients from the (E) TCGA and (F) CGGA databases stratified by IFI30 expression. (G) Time‐dependent receiver operating characteristic curve analysis of the efficiency of the high IFI30 expression group in predicting 1‐, 3‐, and 5‐year OS in the TCGA and CGGA datasets. (H) Univariate analysis and (I) multivariate Cox analyses of clinical prognostic parameters in the TCGA and CGGA datasets. (J) The protein expression of IFI30 in NBT, PA, and different histological grades was detected by WB. (K) The protein expression of IFI30 in different glioma cell lines or glioma stem cells and NHAs was evaluated by WB. All panels show the mean ± SD of three independent experiments; ***p < 0.001, ****p < 0.0001. CGGA, Chinese Glioma Genome Atlas; GBM, glioblastoma; IHC, immunohistochemistry; LSS, low‐grade glioma; NBTs, normal brain tissues; OS, overall survival; PA, paracancerous; TCGA, The Cancer Genome Atlas; WB, Western blotting.

In this study, we found that the mRNA expression of IFI30 in glioma tissues was significantly higher than that in normal brain tissues (NBTs) using RNA‐seq data from the GTEx and TCGA datasets (Figure 1A). The results of GEPIA also showed that the expression of IFI30 was significantly enriched in GBM versus low‐grade glioma, and the expression of IFI30 was higher in glioma tissues than in adjacent tissues (Figure 1B). The immunohistochemistry (IHC) results also revealed that IFI30 expression was increased with increasing WHO grade of glioma and was higher than that in NBTs (Figure 1C,D). Moreover, IDH wild‐type GBM patients were divided into an IFI30 high‐expression group and a low‐expression group according to the median IFI30 expression level in glioma patients from the CGGA and TCGA databases. Kaplan–Meier analysis showed that GBM patients in the low IFI30 expression group survived significantly longer than those in the high expression group (Figure 1E,F). Time‐dependent ROC curve analysis was performed to determine the efficiency of IFI30 expression level in predicting 1‐, 3‐, and 5‐year OS in the TCGA and CGGA datasets (Figure 1G). We also performed univariate Cox (Figure 1H) and multivariate Cox (Figure 1I) which demonstrated that IFI30 can serve as an independent prognostic indicator. Additionally, Western blotting (WB) results showed that the protein levels of IFI30 in fresh glioma tissues were significantly higher than those in NBTs, especially in WHO grade 3 and 4 gliomas (Figure 1J), and the protein levels were higher in most glioma cells than in NHA cells (Figure 1K). In conclusion, IFI30, an independent predictor of glioma prognosis, is overexpressed in glioma, and its expression is positively correlated with tumor grade.

### 3.2 IFI30 regulates the proliferation, migration, and invasion of glioma cells

**FIGURE 2:**
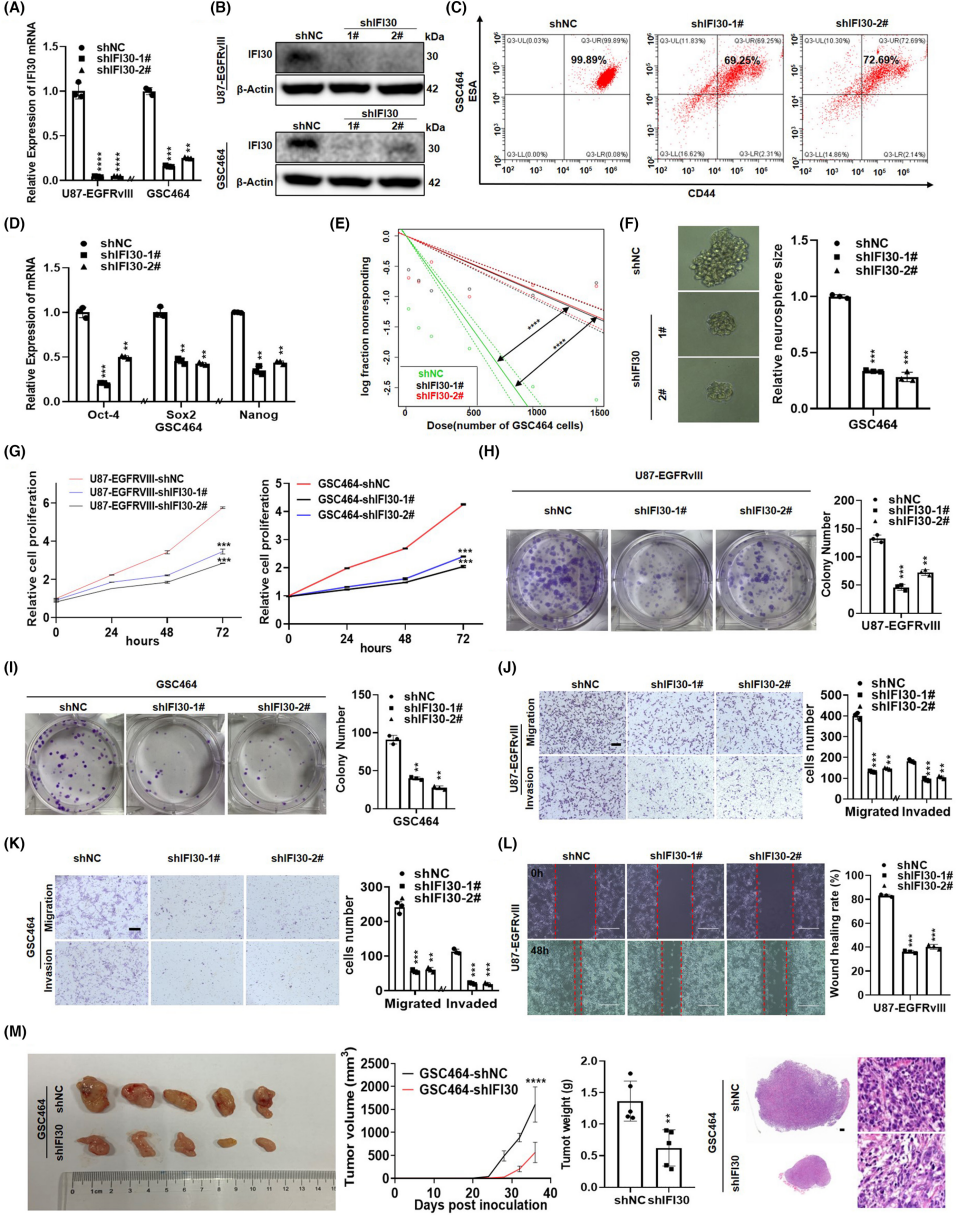
IFI30 regulated the proliferation, migration, and invasion of glioma cells. (A) The mRNA and (B) protein levels of IFI30 in U87‐EGFRvIII and GSC464 cells were transfected with IFI30 lentiviral shRNA. (C) Expression of CD44 and ESA in the control and IFI30 knockdown GSC464 cells according to flow cytometry analyses. (D) The mRNA levels of Oct‐4/Sox2/Nanog in the control and knockdown of the IFI30 in GSC464 cells. (E) A limiting dilution assay was performed in GSC464 cells with or without IFI30 silencing. (F) Representative images of sphere formation capability of GSC464 cells with or without IFI30 inhibition. Bar = 100 μm. Cell proliferation was measured using (G) CCK‐8 and (H, I) colony formation assays. Migration and invasion capabilities were examined by transwell assays in (J) U87‐EGFRvIII and (K) GSC464 cells with or without IFI30 inhibition. Bar = 200 μm. (L) Wound healing analysis was used to determine the migration of U87‐EGFRvIII‐shNC/shIFI30 cells, Bar = 400 μm. The wound closure percentage was calculated by ImageJ software (Rawak Software, Inc.). (M) Representative images of the subcutaneous xenograft tumor size, volume, weight, and H&E staining of GSC464 cells with or without IFI30 inhibition are shown. Bar = 1000 μm. All panels show the mean ± SD of three independent experiments; **p < 0.01, ***p < 0.001, ****p < 0.0001.

Given the upregulation of IFI30 in glioma and its ability to predict survival, we next explored the biological function of IFI30. We constructed U87‐EGFRvIII and GSC464 cells with stable IFI30 knockdown by lentiviral shRNA. RT–qPCR and WB results showed that the IFI30 expression level was reduced by at least 60% (Figure 2A,B). The molecular surface markers CD44 and ESA were assessed to identify whether the cells were tumor stem cells. Flow cytometry showed that the expression of CD44 and ESA in GSC464 cells was significantly higher than that in GSC464‐shIFI30 cells (Figure 1C). In addition, RT–qPCR (Figure 1D) and WB (Figure S1A) showed that the expression of the stem cell‐related transcription factors Oct‐4/Sox2/Nanog in GSC464 cells was significantly higher than that in GSC464‐shIFI30 cells. A limiting dilution assay was performed to examine the self‐renewal ability of glioma stem cells (Figure 2E). GSC464‐shIFI30 cells gave rise to fewer and smaller spheres (Figure 2F). Moreover, we examined the effect of IFI30 downregulation on cell viability with CCK‐8 and colony formation assays. We found that silencing IFI30 significantly decreased the viability of glioma cells (Figure 2G–I). Next, we performed wound healing, migration, and invasion assays and found that cell migration was observably reduced after IFI30 knockdown (Figure 2J–L). To further prove the antitumor effect of IFI30 silencing on glioma in vivo, a xenograft model was established by subcutaneous injection of GSC464‐shNC and GSC464‐shIFI30 cells. As shown, knockdown of IFI30 significantly reduced tumor volume and weight compared with those in the control group (Figure 2M). In contrast, we overexpressed IFI30 in glioma cells (Figure S2A,B) to further investigate the regulatory role of IFI30 in glioma progression. RT–qPCR (Figure S1B) and WB (Figure S1C) showed that the expression of the stem cell‐related transcription factors Oct‐4/Sox2/Nanog in GSC267 cells was significantly lower than that in GSC267‐OE‐IFI30 cells. Limiting dilution assays showed that GSC267‐OE‐IFI30 cells harbored more and larger spheres (Figure S2C,D). Flow cytometry showed that the expression of CD44 and ESA in the GSC267 cells was significantly lower than that in GSC267‐OE‐IFI30 cells (Figure S2E). GSC267‐NC and GSC267‐OE‐IFI30 cells (5 × 10^5^ cells/mouse, six mice/group) were injected intracerebrally into mice to construct a mouse xenograft model. Overexpression of IFI30 significantly increased the tumor volume compared with that in the control group. Kaplan–Meier survival analysis demonstrated that the survival time of xenograft mice was noticeably shortened after IFI30 overexpression (Figure S2F). CCK‐8 and colony formation assays confirmed the findings that IFI30 overexpression could induce the proliferation of glioma cells compared to that in the NC group (Figure S1G–I). We observed that IFI30 overexpression also markedly promoted cell migration and invasion (Figure S1J–L). Taken together, these data suggest that IFI30 promotes the proliferation, migration, and invasion of glioma cells.

### 3.3 IFI30 promotes the EMT‐like process and activates the EGFR/AKT/GSK3β/β‐catenin signaling pathway in glioma cells in vitro and in vivo

**FIGURE 3:**
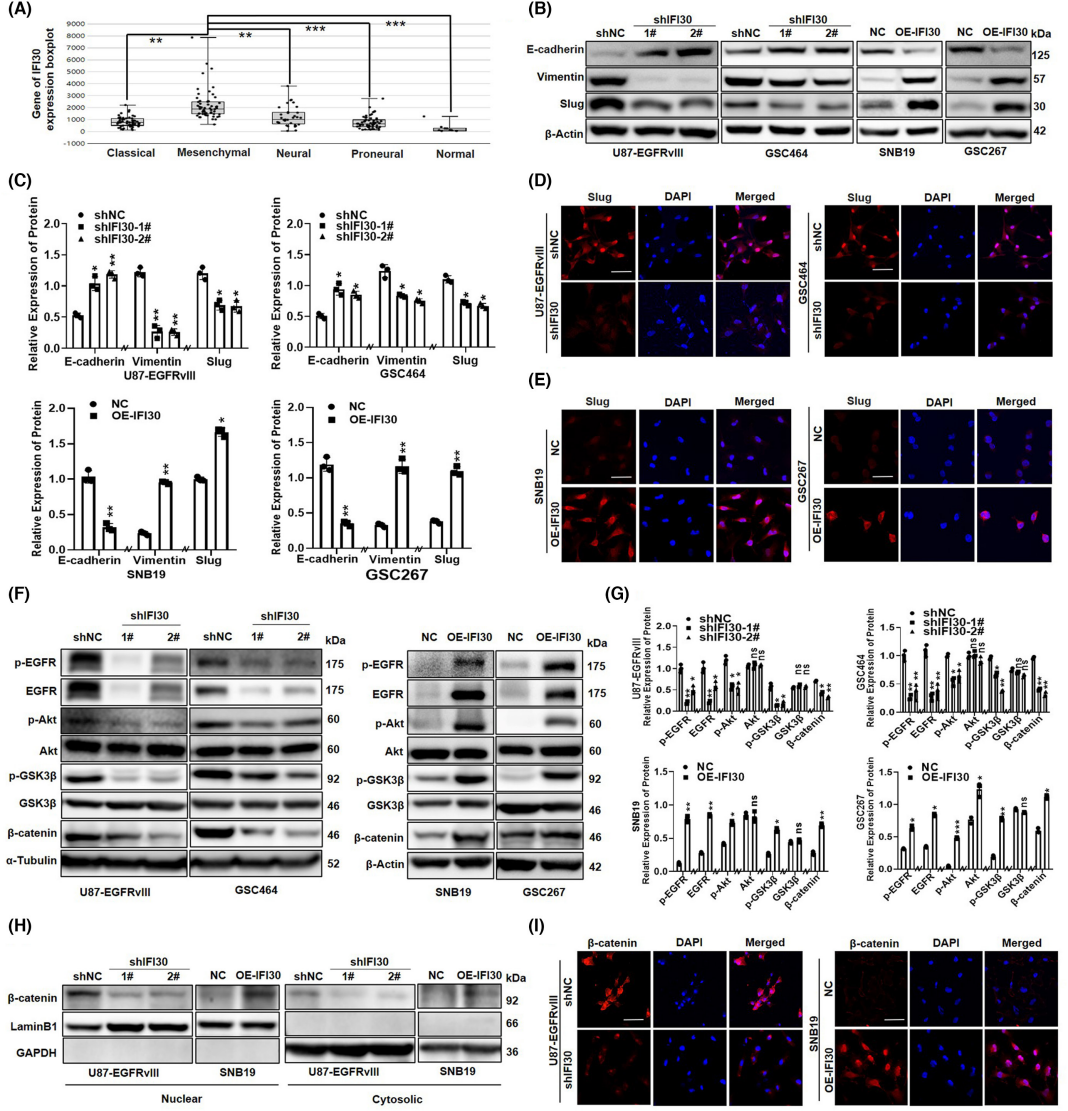
IFI30 Promoted the EMT‐like process and activated the EGFR/AKT/GSK3β/β‐catenin signaling pathway in glioma cells in vitro and in vivo. (A) Distribution of IFI30 expression in different glioma molecular subtypes based on RNA‐seq data in TCGA. (B) Western blotting was used to detect the expression of Slug, vimentin, and E‐cadherin after knocking down or overexpressing IFI30 in glioma cells. (C) Quantification of the protein expression after knocking down or overexpressing IFI30 in glioma cells was performed with ImageJ software; β‐Actin was used as a loading control. Slug immunofluorescence staining images of (D) IFI30‐silenced or (E) IFI30‐overexpressing cells were captured with confocal microscopy. Bar = 100 μm. (F) Western blotting was used to detect the expression of EGFR, p‐EGFR, Akt, p‐Akt, GSK3β, p‐GSK3β, and β‐catenin after knocking down or overexpressing IFI30 in glioma cells. (G) Quantification of protein expression after knockdown or overexpression of IFI30 in glioma cells; α‐tubulin, and β‐actin were used as loading controls. (H) The cytoplasmic and nuclear fractions of U87‐EGFRvIII and SNB19 cells were extracted separately, and then the expression of β‐catenin was detected by western blotting. GAPDH was used as the cytoplasmic loading control, and Lamin B1 was used as the nuclear loading control. (I) Immunofluorescence staining confirmed the distribution of β‐catenin inside the cell. DAPI was used for nuclear staining; Bar = 100 μm. All panels show the mean ± SD of three independent experiments; *p < 0.05, **p < 0.01, ns, no significance. DAPI, 4′,6‐diamidino‐2‐phenylindole; EMT, epithelial–mesenchymal transition.

To explore the role of IFI30 in inducing the EMT‐like process, we analyzed glioma subtype‐specific IFI30 expression in the TCGA. IFI30 expression was extremely high in the mesenchymal subtype compared with the other subtypes (Figure 3A). Moreover, we showed that IFI30 expression positively correlated with the expression of multiple EMT‐related markers, such as Snail1, Snail2, Twist1, and vimentin (Figure S3A). In addition, the results of RT–qPCR showed that knockdown of IFI30 significantly reduced the mRNA expression of Slug and vimentin, but increased the expression of E‐cadherin (Figure S3B). Overexpression of IFI30 enhanced the expression of EMT markers in SNB19 and GSC267 cells (Figure S3C). Moreover, the WB results showed that IFI30 knockdown significantly reduced the expression of vimentin and Slug but increased E‐cadherin expression in U87‐EGFRvIII and GSC464 cells. Overexpression of IFI30 enhanced the expression of EMT markers in SNB19 and GSC267 cells (Figure 3B,C). Furthermore, immunofluorescence staining revealed that Slug and vimentin expression was obviously reduced in IFI30 knockdown cells (Figure 3D; Figure S3D), but E‐cadherin expression was increased. We obtained the opposite result in IFI30‐overexpressing cells (Figure 3E; Figure S3E). EGFR signaling activation induces the expression of multiple EMT markers and promotes the migration and proliferation of glioma.^31^ To gain insight into the molecular mechanism underlying IFI30‐mediated EMT‐like, we analyzed the correlation between IFI30 and EGFR. The results showed that the mRNA expression level of EGFR was significantly decreased in IFI30‐silenced glioma cells but increased in IFI30‐overexpressing cells (Figure S3F). Moreover, we analyzed the pathways enriched in genes that were positively associated with IFI30 expression in the TCGA and CGGA datasets. The GSEA results showed that AKT was significantly correlated with IFI30 expression in glioma (Figure S3G). Then, we performed WB to detect the expression of EGFR, p‐EGFR (Tyr1068), AKT, p‐AKT (Ser473), GSK3β, p‐GSK3β (Ser9), and β‐catenin. Our results showed that knocking down IFI30 significantly inhibited the expression of p‐EGFR, EGFR, p‐AKT, p‐GSK3β, and β‐catenin, but the expression of total AKT and GSK3β showed no significant change (Figure 3F,G). In addition, we found that IFI30 knockdown reduced the levels of cytosolic and nuclear β‐catenin in glioma cells, while IFI30 overexpression increased the expression of β‐catenin (Figure 3H,I). All the above results indicate that overexpression of IFI30 significantly enhanced the expression of Ki‐67, EGFR, p‐AKT, p‐GSK3β, and β‐catenin in vitro.

### 3.4 The EGFR/AKT/GSK3β/β‐catenin signaling pathway is involved in the IFI30‐mediated EMT‐like process

**FIGURE 4:**
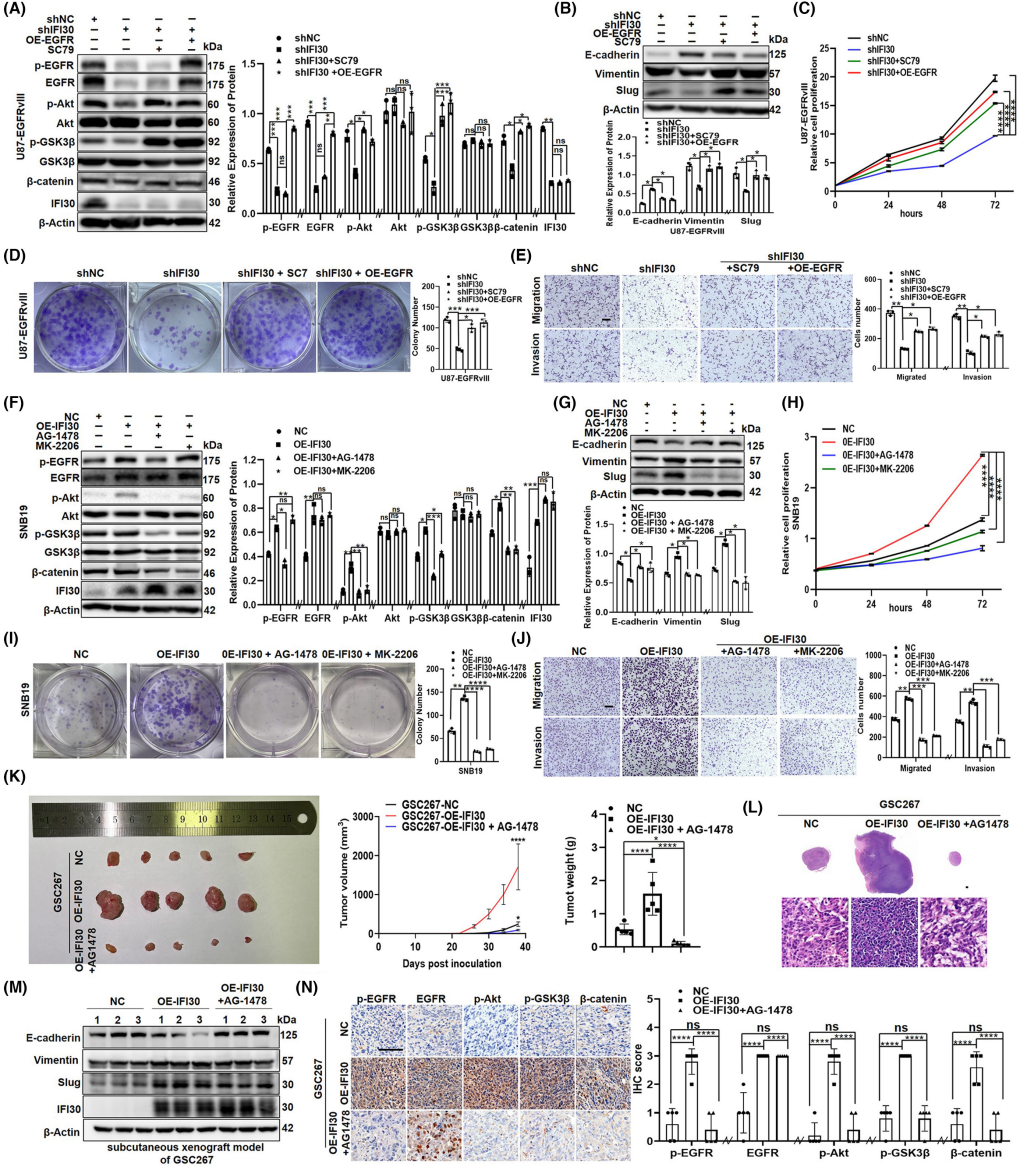
The EGFR/AKT/GSK3β/β‐catenin signaling pathway was involved in the IFI30‐mediated EMT‐like process. IFI30 knockdown cells were treated with EGFR plasmid and SC79. (A) Western blotting was used to detect the expression of EGFR, p‐EGFR, Akt, p‐Akt, GSK3β, p‐GSK3β, β‐catenin, IFI30, vimentin, Slug, and E‐cadherin. (B) Quantification of the protein expression was performed with ImageJ software; β‐Actin was used as a loading control. (C) CCK‐8, (D) colony formation, migration, and (E) invasion assays were used to detect the proliferation, migration, and invasion abilities. The IFI30‐overexpressing cells were treated with AG‐1478 and MK‐2206. (F) Western blotting was used to detect the expression of EGFR, p‐EGFR, Akt, p‐Akt, GSK3β, p‐GSK3β, β‐catenin, IFI30, vimentin, Slug, and E‐cadherin. (G) Quantification of the protein expression was performed with ImageJ software; β‐Actin was used as a loading control. (H) CCK‐8, (I) Colony formation, migration, and (J) invasion assays were used to detect the proliferation, migration, and invasion abilities. (K) Representative images and H&E staining of GSC267‐NC and GSC267‐OF‐IFI30 cell subcutaneous xenografts treated with or without AG1478 are shown, Bar = 1000 μm. (L) Representative p‐EGFR, EGFR, p‐AKT, p‐GSK3β, and β‐catenin staining images of tumor sections from GSC267‐NC and GSC267‐OF‐IFI30 with or without AG‐1478 cell subcutaneous xenografts with or without AG1478. Bar = 100 μm. All panels show the mean ± SD of three independent experiments; *p < 0.05, **p < 0.01, ***p < 0.001, ****p < 0.0001, ns, no significance.

To assess whether the EGFR/AKT/GSK3β/β‐catenin signaling pathway is essential for the IFI30‐mediated EMT‐like process, we selected an EGFR overexpression plasmid and AKT activator (SC79) to complete the rescue experiment. Then, our WB (Figure 4A,B) results showed that the EGFR plasmid enhanced the IFI30 knockdown‐induced suppression of EGFR, p‐EGFR, p‐AKT, p‐GSK3β, β‐catenin, vimentin, and Slug expression and reduced the IFI30 knockdown‐induced promotion of E‐cadherin expression in U87‐EGFRvIII cells, but the expression of IFI30, total AKT, and total GSK3β showed no significant change; SC79 enhanced the expression of p‐AKT, p‐GSK3β, β‐catenin, vimentin, and Slug and reduced E‐cadherin expression, but the expression of IFI30, total EGFR, p‐EGFR, total AKT, and total GSK3β showed no significant change. Immunofluorescence staining showed the same results (Figure S4C). CCK‐8, colony formation, wound healing, migration, and invasion assays showed that the EGFR plasmid and SC79 increased the IFI30 knockdown‐induced suppression of proliferation, migration, and invasion (Figure 4C–E; Figure S4A). In contrast, treatment with an EGFR inhibitor (AG‐1478) attenuated IFI30 overexpression‐induced promotion of p‐EGFR, p‐AKT, p‐GSK3β, β‐catenin, vimentin, and Slug expression and IFI30 overexpression‐induced suppression of E‐cadherin expression in SNB19 cells, but the expression of IFI30, total EGFR, total AKT, and total GSK3β showed no significant change; AKT inhibitor (MK‐2206) attenuated IFI30 overexpression‐promotion of p‐AKT, p‐GSK3β, β‐catenin, vimentin, and Slug, and IFI30 overexpression‐induced suppression of E‐cadherin expression, but the expression of IFI30, total EGFR, p‐EGFR, total AKT, and total GSK3β showed no significant change (Figure 4F,G). The CCK‐8, colony formation, wound healing, migration, and invasion results showed that AG‐1478 and MK‐2206 treatment decreased the proliferation‐, migration‐, and invasion‐promoting effects of IFI30 in SNB19‐OE‐IFI30 cells (Figure 4H–J; Figure S4B). Immunofluorescence staining showed the same results (Figure S4D). To further prove that EGFR plays a role in the IFI30‐mediated EMT‐like process in vivo, a xenograft model was established by subcutaneous injection of GSC267‐NC and GSC267‐OE‐IFI30 cells with or without AG‐1478 treatment. As shown, overexpression of IFI30 significantly increased tumor volume and weight compared with that in the control and AG‐1478 groups (Figure 2K,L). WB showed that AG‐1478 significantly decreased the expression of EMT‐like markers (Figure 2M) and IHC staining showed that AG‐1478 significantly decreased the expression of Ki‐67, EGFR, p‐AKT, p‐GSK3β, and β‐catenin in vivo (Figure 2N). These data further confirm that IFI30 promotes the EMT‐like process of glioma cells by regulating the EGFR/AKT signaling pathway.

### 3.5 IFI30 enhances the resistance of glioma cells to TMZ chemotherapy in vitro and in vivo

**FIGURE 5:**
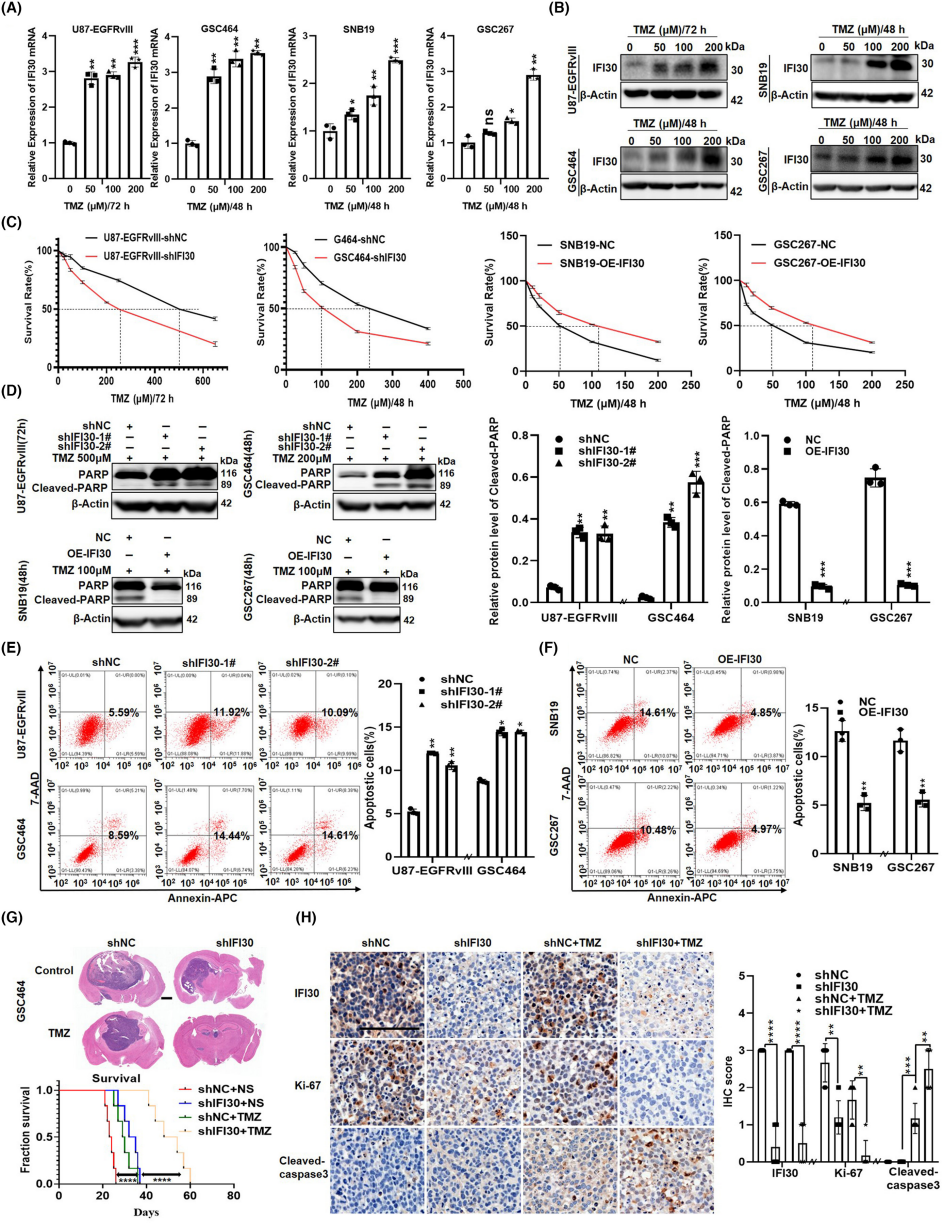
IFI30 enhanced the resistance of glioma cells to TMZ chemotherapy in vitro and in vivo. (A) IFI30 mRNA and (B) protein expression was examined with RT–PCR and WB in U87‐EGFRvIII, GSC464, SNB19, and GSC267 cells treated with different doses of TMZ. (C) CCK‐8 assay was used to detect the survival rate of U87‐EGFRvIII/GSC464‐shNC and shIFI30, SNB19/GSC267‐NC, and SNB19/GSC267‐OE‐IFI30 cells treated with different concentrations of TMZ. (D) Cleaved PARP was examined with WB in U87‐EGFRvIII/GSC464‐shNC and shIFI30, SNB19/GSC267‐NC, and ‐OE‐IFI30 cells treated with TMZ. Quantification of the protein expression was performed with ImageJ software; β‐Actin was used as a loading control. Cell apoptosis was examined by flow cytometry in (E) U87‐EGFRvIII/GSC464‐shNC and shIFI30 and (F) SNB19/GSC267‐NC and ‐OE‐IFI30 cells treated with TMZ. Annexin‐APC staining was used to detect apoptosis. (G) Representative H&E staining of brain sections on day 20 after intracranial inoculation of GSC464‐shNC or GSC464‐shIFI30 in nude mice treated with NS or TMZ separately. 5 × 10^5^ cells/mouse, 6 mice/group. Kaplan–Meier survival curve of mice. Statistical comparison was performed using a log‐rank test. (H) Representative IFI30, Ki67, and cleaved caspase‐3 staining images of tumor sections from GSC464‐shNC or GSC464‐shIFI30 mice treated with NS or TMZ are shown. Bar = 100 μm. All panels show the mean ± SD of three independent experiments; *p < 0.05, **p < 0.01, ***p < 0.001, ****p < 0.0001. TMZ, temozolomide; WB, western blotting.

First, we found that IFI30 mRNA levels were significantly elevated in response to TMZ in glioma cells (Figure 5A). Moreover, TMZ treatment also upregulated IFI30 protein expression in a dose‐dependent manner (Figure 5B). To explore whether ifi30 is involved in the TMZ resistance process, U87‐EGFRvIII TR, and SNB19 TR cell lines were established by TMZ treatment within 3 months, and WB analysis revealed increased IFI30 levels upon TMZ resistance compared to the vehicle control (Figure S5A). RT–qPCR showed the same results (Figure S5B). We used the CCK‐8 assay to detect the survival rate of IFI30‐silenced glioma cells and IFI30‐overexpressing cells treated with different concentrations of TMZ. The results showed that inhibition of IFI30 enhanced the efficacy of TMZ and decreased the cell survival rate. Overexpression of IFI30 dramatically increased the survival rate of glioma cells (Figure 5C). WB analysis revealed increased cleaved PARP levels upon inhibition of IFI30 compared to the vehicle control, while overexpression of IFI30 dramatically decreased the cleaved PARP levels, confirming apoptosis regulation (U87‐EGFRvIII 500 μM/72 h and GSC464 200 μM/48 h, SNB19 100 μM/48 h, and GSC267 100 μM/48 h, based on the IC_50_ of temozolomide) (Figure 5D). Moreover, inhibition of IFI30 promoted the apoptosis of glioma cells treated with various concentrations of TMZ, as detected by flow cytometry (Figure 5E), while overexpression of IFI30 suppressed the apoptosis of glioma cells treated with certain TMZ concentrations (Figure 5F). Next, we performed an in vivo experiment. Our intracranial xenograft model in nude mice showed that IFI30 knockdown in GSC464 cells suppressed tumorigenesis, and the GSC464‐shIFI30 group treated with TMZ developed much smaller tumors than the GSC464‐shNC central group treated with TMZ, 5 × 10^5^ cells/mouse, six mice/group. Survival analysis showed that IFI30 knockdown combined with TMZ treatment significantly prolonged survival compared with IFI30 knockdown alone or TMZ treatment alone (Figure 5G). IHC staining showed that IFI30 was upregulated in GSC464‐shNC and GSC464‐shIFI30 cells treated with TMZ. In addition, the GSC464‐shIFI30 group had fewer Ki67‐positive cells than the shNC group with or without TMZ treatment but had more Caspase 3‐positive cells than the corresponding control groups with TMZ treatment (Figure 5H). Taken together, these findings suggest that IFI30 can enhance TMZ resistance and decrease the cytotoxic effect of TMZ therapy in vitro and in vivo.

### 3.6 IFI30‐mediated EMT plays a role in TMZ resistance in glioma cells

**FIGURE 6:**
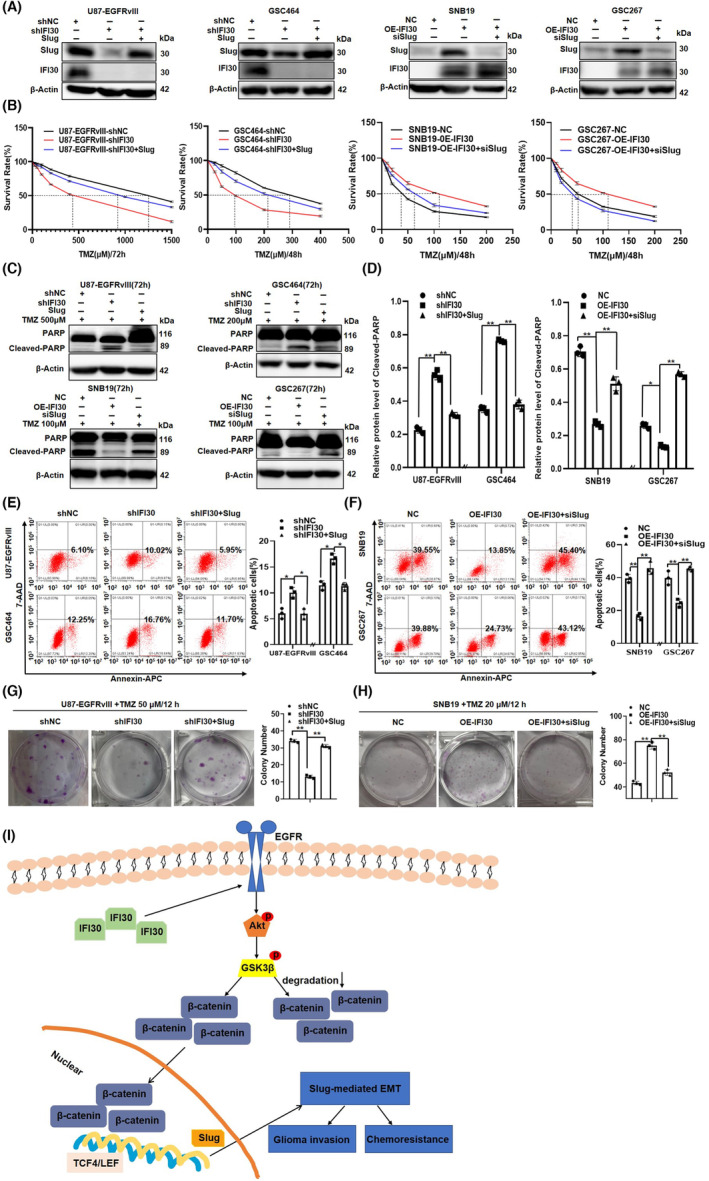
IFI30‐Mediated EMT played a role in TMZ resistance in glioma cells. We constructed specific cell lines by transiently transfecting Slug overexpression plasmids into shIFI30 cells and transiently transfecting Slug‐silencing siRNA into OE‐IFI30 cells for the rescue assay. (A) Western blotting was used to detect the expression of IFI30 and Slug in these cells. (B) A CCK‐8 assay was used to detect the survival rate of these cells treated with different concentrations of TMZ. (C) Western blotting was used to detect the expression of IFI30 and Slug in these cells. (D) Quantification of protein expression was performed with ImageJ software; β‐Actin was used as a loading control. (E, F) Cell apoptosis was examined by flow cytometry in cells treated with TMZ. Annexin‐APC staining was used to detect apoptosis. (G, H) Colony formation assays were used to detect the proliferation and colony formation of two glioma cell lines treated with TMZ. (I) A schematic representation illustrating the role of IFI30 in regulating EMT and chemoresistance in glioma cells. Based on the findings of this study, IFI30 could activate the EGFR/AKT/GSK3β/β‐catenin signaling pathway and enhance Slug expression, which eventually promotes EMT and chemoresistance. All panels show the mean ± SD of three independent experiments; *p < 0.05, **p < 0.01. EMT, epithelial–mesenchymal transition; TMZ, temozolomide.

Accumulating evidence has shown that the EMT process is closely associated with chemotherapy resistance in glioma cells.^32^ Slug, a key regulator of the EMT‐like process and a zinc‐finger transcription factor, represses E‐cadherin transcription via the E‐box elements in the proximal E‐cadherin promoter in EMT progression.^33^ To confirm the role of the EMT‐like process mediated by IFI30 in inducing TMZ resistance, we constructed specific cell lines by transiently transfecting Slug overexpression plasmids into shIFI30 cells for the rescue assay. The WB results showed significant upregulation of slug, but the expression of IFI30 was not affected. In contrast, we also transiently transfected Slug‐silencing siRNA into OE‐IFI30 cells. The WB results showed significant downregulation of slug, but the expression of IFI30 showed no difference (Figure 6A). We used certain TMZ concentrations to treat these glioma cells (U87‐EGFRvIII 500 μM/72 h, GSC464 200 μM/72 h, SNB19 100 μM/72 h, and GSC267 100 μM/72 h, based on the IC_50_ of temozolomide); the results of the CCK‐8 assay showed that overexpression of Slug prevented the IFI30 knockdown‐induced enhancement of the efficacy of TMZ and increased the cell survival rate. Inhibition of Slug reversed the IFI30 overexpression‐induced increase in the survival rate of glioma cells (Figure 6B). Moreover, WB analysis revealed that Slug overexpression markedly attenuated the IFI30 knockdown‐induced increase in cleaved PARP levels and that Slug inhibition reversed the IFI30 overexpression‐induced decrease in cleaved PARP levels (Figure 6C,D). In addition, Slug overexpression suppressed the IFI30 knockdown‐induced increase in apoptosis after treatment with certain TMZ concentrations, as detected by flow cytometry (Figure 6E), while Slug inhibition reversed the IFI30 overexpression‐induced decrease in apoptosis after treatment with TMZ (Figure 6F). Next, colony formation assays were used to detect the proliferation of glioma cells treated with TMZ (U87‐EGFRvIII 50 μM/12 h and GSC464 20 μM/12 h). IFI30 knockdown inhibited U87‐EGFRvIII cell proliferation and colony formation ability under TMZ treatment conditions. However, Slug overexpression dramatically reversed the IFI30 knockdown‐induced inhibition of proliferation and colony formation (Figure 6G). Consistent with this, IFI30 overexpression promoted SNB19 cell proliferation and colony formation ability under both normal and TMZ‐treated conditions, and inhibition of Slug prevented the IFI30 overexpression‐induced promotion of proliferation and colony formation (Figure 6H). Taken together, these results suggest that IFI30‐mediated EGFR/AKT/GSK3β/β‐catenin signaling not only promotes the EMT‐like phenotype by up‐regulating the expression of Slug, but also subsequently enhanced invasion and chemoresistance of glioma cells (Figure 6I).

## 4 DISCUSSION

In this study, we investigated the biological function of IFI30 in both normal and TMZ‐treated glioma cells. We demonstrated that IFI30 was highly expressed in glioma tumors and cell lines, and its expression was negatively correlated with the prognosis of patients. Functionally, IFI30 promoted the proliferation, migration, and invasion of glioma cells in vitro, and its expression significantly enhanced the growth of glioma in vivo. We further demonstrated that the EGFR/AKT/GSK3β/β‐catenin axis played a role in IFI30‐promoted the EMT‐like phenotype, which is a well‐recognized mechanism in the diffuse infiltration of glioma cells. Moreover, we also observed that IFI30 expression was significantly induced by TMZ treatment at both the protein level and the RNA level. In addition, in vivo and in vitro studies showed that IFI30 silencing suppressed tumor progression and enhanced the therapeutic effect of TMZ treatment. Importantly, we found that IFI30 regulated the chemoresistance of glioma cells to TMZ directly via the expression of the transcription factor Slug, a key regulator of EMT. Thus, these results indicate that IFI30 not only promotes glioma tumor progression but also promotes TMZ chemoresistance in glioma.

In 1988, Luster et al. isolated the ISG IFI30 from a cDNA clone of a monocytic cell line for the first time and found that it was continuously expressed in hematopoietic cell lines, while in nonhematopoietic cell lines, it could be expressed under the induction of IFN‐γ.^27^ With the in‐depth study of IFI30, it has been found that it also plays an important role in the occurrence and development of various cancers, such as malignant melanoma, hematopoietic malignancies, and breast cancer.^30^

Bioinformatics analysis in this study of an online public database indicated that IFI30 was highly expressed in glioma samples. Moreover, high expression of IFI30 was significantly associated with worse OS. We further examined IFI30 protein expression levels in clinical glioma samples and obtained the same results, confirming that IFI30 was highly expressed in glioma tissues and was associated with poor outcomes. This is consistent with the report of Zhu et al., Liu et al., and Jiang et al.^25^, ^34^, ^35^ Then, both loss‐of‐function and gain‐of‐function studies were performed in glioma cell lines and glioma stem cells and confirmed that IFI30 facilitated the proliferation of glioma cells in vitro and in vivo. Interestingly, the results in glioma were contradictory to recent findings in other cancers. Furthermore, previous studies showed that IFI30 catalyzes the reduction of disulfide bonds in antigen proteins in APCs to present antigenic epitopes to T cells.^28^ This function enhances the antitumor immunity of T cells.^25^ Thus, the elevated expression of IFI30 might improve the prognosis of patients with DLBCL and breast cancer.^30^, ^36^ However, the absence of the lymphatic system and the existence of the blood–brain barrier in the CNS prevent IFI30 from inducing antitumor immunity in glioma.^7^ Moreover, the mechanism underlying the posttranscriptional regulation of IFI30 expression, which might also be responsible for the above phenomenon, has yet to be elucidated.

EMT is pivotal for embryonic development and wound healing processes, and its occurrence in cancer is known to aggravate cell invasion, migration, and drug resistance.^37^ A recent study using whole‐exon and whole‐transcriptome sequencing of patients with metastatic tumors demonstrated a strong correlation between the expression of interferon‐induced genes and EMT.^23^ Although there are some different features and behavioral patterns between the EMT‐like process in neuroepithelial tumors and the classical EMT process in traditional epithelial tumors, many mesenchymal markers play important roles in glioma malignancy.^38^ Consistent with these findings, we found that IFI30 expression was mainly enriched in the mesenchymal subtype and correlated with multiple EMT‐related markers in TCGA datasets. Additionally, knocking down or overexpressing IFI30 dramatically altered the expression of Slug, vimentin, and E‐cadherin. The alteration of the EMT‐like process is accompanied by a decrease or increase in the expression of EMT‐like transcription factors and a remarkable decline or enhancement in cell invasiveness. Our study provided solid evidence that IFI30 might be a novel regulator of the EMT‐like process in glioma and explains why the high expression of IFI30 in glioma can promote tumor growth.

EGFR is a transmembrane glycoprotein receptor tyrosine kinase, and its gene EGFR is frequently altered in GBM.^32^ Over 60% of GBMs have EGFR amplification, and half of these harbor mutations, the most frequent being the gain‐of‐function mutation EGFRvIII, in which deletion of exons 2–7 generates an in‐frame truncation in the extracellular domain that leads to constitutive kinase activity.^39^ EGFR and EGFRvIII have been linked to the invasive behavior of GBMs together with increased angiogenesis, which has an established role in maintenance of EMT.^40^ Considering the above characteristics, EGFR‐targeted therapy has been recognized as a promising therapeutic strategy.^41^ However, almost all relevant clinical trials on various EGFR tyrosine kinase inhibitors (TKIs) for primary and recurrent glioma patients have yielded little success.^42^ Their failure was unexpected considering the prevalence of EGFR amplification in glioma, and the most significant factor contributing to this failure was inadequate target inhibition and drug resistance.^43^ Therefore, it is very important to find the upstream target that directly regulates EGFR. One of the important findings of this study is that IFI30 can directly regulate the expression of EGFR. GSEA indicated that AKT was significantly correlated with IFI30 expression in glioma. Moreover, EGFR stimulates cell migration through receptor phosphorylation and subsequent activation of downstream signaling pathways, including the AKT and MAPK pathways, to support glioma growth.^31^ GSK3β, an important AKT signaling substrate, displays a regulatory function on mitochondrial activities. Inhibition of GSK3β via its phosphorylation at Ser9 by activated AKT signaling can lead to β‐catenin stabilization and its translocation into the nucleus for gene transcription.^44^ Therefore, it is reasonable to suggest that IFI30 may regulate the EGFR/AKT/GSK3β/β‐catenin signaling pathway. To assess whether the EGFR/AKT/GSK3β/β‐catenin signaling pathway is essential for the IFI30‐mediated EMT‐like process, EGFR plasmid, activator of AKT, inhibitor of EGFR and inhibitor of AKT were applied. The results of WB and restoration of function experiments confirmed that IFI30 promotes the EMT‐like process by regulating the EGFR/AKT/GSK3β/β‐catenin signaling pathway.

The development of drug resistance, specifically resistance to TMZ, is a major challenge in GBM treatment.^45^ Natsume et al. found that IFN‐β can promote the transcription and expression of the p53 gene by activating the ISGF3 pathway, thereby downregulating the expression of MGMT. Then, they treated nude mouse xenografts with IFN‐β combined with TMZ and achieved significant efficacy.^46^ Ji et al. reported that IFN‐γ‐induced GBP1 was highly expressed in gliomas, making glioma cells resistant to TMZ and radiotherapy.^47^ Menyhárt et al. revealed that IFN‐α/β‐induced MX1 expression was upregulated in glioma patients after TMZ treatment and that MX1 expression was negatively correlated with MGMT expression.^48^ An increasing number of studies have shown that ISGs can regulate sensitivity to TMZ. Consistent with these findings, we found that TMZ treatment induced the upregulation of IFI30 in glioma cells and that the expression of IFI30 significantly promoted the proliferation and suppressed the apoptosis of glioma cells treated with TMZ. Both in vitro and in vivo experiments showed that IFI30‐overexpressing cells were more resistant to TMZ than control cells, and IFI30 overexpression resulted in fewer apoptotic cells. Overall, IFI30 enhanced TMZ resistance and decreased the cytotoxic effect of TMZ therapy on glioma cells.

Numerous studies have reported that drug resistance in tumors is related to the EMT process. Jakobsen et al. showed that EMT status influenced EGFR‐TKI resistance via the Wnt or Notch pathway in EGFR‐mutant NSCLC cancer.^49^ Cheng et al. suggested that GBP1 regulated erlotinib resistance via PGK‐mediated EMT signaling. Babu et al. revealed an anticancer and temozolomide‐sensitizing effect of rabeprazole by repressing EMT.^50^ Therefore, we further evaluated the importance of the EMT‐like process in mediating TMZ resistance via IFI30 and discovered that IFI30 strongly depends on the EMT‐like pathway to regulate TMZ resistance. Additionally, the overexpression and silencing of Slug in shIFI30 and OE‐IFI30 glioma cells led to a change in TMZ sensitivity. All the above results suggest that the activation of EMT‐like by IFI30 promotes TMZ resistance in glioma. In addition, GSCs are responsible for drug resistance and tumor recurrence in patients with glioma.^10^ We also examined the stem cell component through a sphere formation assay (Figure S6A) and CD44/ESA staining (Figure 6B) of TMZ‐treated cells, and the results showed that IFI30 can enhance the stemness and TMZ resistance of glioma, which represents a new direction for future studies and current studies that are ongoing in our laboratory. There are still many shortcomings in our research, especially the absence of small molecule inhibitors of IFI30, which are not currently available domestically or internationally. We plan to collaborate with relevant biological companies to develop small molecule inhibitors of IFI30 in future research.

In conclusion, in the present study, we have demonstrated that the transcription factor IFI30 can serve as an indicator of a poor prognosis in glioma and that IFI30 promotes the EMT‐like process of glioma cells by regulating the EGFR/AKT/GSK3β/β‐catenin signaling pathway, which subsequently enhances the invasion and chemoresistance of glioma cells. Thus, we conclude that IFI30 is a regulator of the EMT‐like process and acts not only as a prognostic marker but also as a novel biomarker of the response to TMZ in glioma.

## AUTHOR CONTRIBUTIONS

Ying Chen performed the experiments and wrote the first draft of the article. Hui Xu designed the study. Pei Yu analyzed the data. Qing Wang, Fufu Ji, and Chunwang Wu carried out investigation. Qing Lan was involved in writing—reviewing and supervision.

## FUNDING INFORMATION

This work was supported by the National Natural Science Foundation of China (NSFC: 81272799 to Q.L.), the project funded by Suzhou Key Laboratory of Minimally Invasive Neurosurgery (SZ2021262 to Q.L.), and the project funded by Suzhou City Clinical Medicine Center (SZLCYXZXJ202103 to Q.L.).

## CONFLICT OF INTEREST STATEMENT

The authors have declared that no competing interest exists.

## Supporting information


Data S1.

Data S2.

## Data Availability

The data that support the findings of this study are available from the corresponding authors upon reasonable request.
