# Supplementary material for: Interferon‐γ inducible protein 30 promotes the epithelial–mesenchymal transition‐like phenotype and chemoresistance by activating EGFR/AKT/GSK3β/β‐catenin pathway in glioma
Source: CNS Neurosci Ther. 2023 Jul 5;29(12):4124–38. doi: 10.1111/cns.14334 (PMC10651985; doi:10.1111/cns.14334)
Supplement: Supplementary file 1 — Data S1. [file CNS-29-4124-s001.docx]

**Supplementary Materials**

*for*

**Interferon-γ inducible protein 30 promotes the epithelial mesenchymal transition-like phenotype and chemoresistance by activating EGFR/AKT/GSK3β/β-catenin pathway in glioma**

Ying Chen, MS^1^; Hui Xu, MD^1^; Pei Yu, MS^1^; Qing Wang, MD^1^; Shenggang Li, MD^1^; Fufu Ji, MS^1^; Chunwang Wu, MS^1^; Qing LAN, MD^1^.

1. Department of Neurosurgery, The Second Affiliated Hospital of Soochow University, Suzhou, P.R. China;

To whom correspondence should be addressed to

Qing LAN, MD; Director, Department of Neurosurgery,

the Second Affiliated Hospital of Soochow University, Suzhou, Jiangsu 215004, PR China.

Tel: +86 51267784087, e-mail: [szlq006@163.com](mailto:szlq006@163.com).

ORCID ID: 0000-0002-8468-4942

**1. Table S1. Univariate analysis and multivariate COX analysis of clinical prognostic parameters in CGGA dataset.**

| **Factors** | **Univariate analysis** | |  | **Multivariate analysis** | |
| --- | --- | --- | --- | --- | --- |
|  | **Hazard ratio (95% CI)** | ***P* value** |  | **Hazard ratio (95% CI)** | ***P* value** |
| PRS_type | 2.123 (1.818-2.478) | 1.79E-21 |  | 1.930 (1.643-2.268) | 1.35 E-15 |
| Histology | 4.487 (3.695-5.449) | 7.38E-52 |  | 0.668 (0.429-1.040) | 0.074 |
| Grade | 2.883 (2.526-3.291) | 1.44E-55 |  | 2.748 (2.011-3.755) | 2.23E-10 |
| Gender | 1.044 (0.866-1.258) | 0.66 |  | 1.055 (0.871-1.276) | 0.58 |
| Age | 1.624 (1.345-1.960) | 4.49E-07 |  | 1.248 (1.022-1.524) | 0.03 |
| Radio | 0.929 (0.720-1.199) | 0.57 |  | 0.878 (0.671-1.149) | 0.34 |
| Chemo | 0.678 (1.328-2.044) | 5.71 |  | 0.679 (0.534-0.863) | 0.001 |
| IDH_mutation | 0.317 (0.262-0.384) | 3.84E-32 |  | 0.642 (0.505-0.817) | 0.0003 |
| 1p19q_codeletion | 0.231 (0.169-0.315) | 2.08E-20 |  | 0.406 (0.290-0.567) | 2.6854E-07 |
| IFI30 expression | 1.348 (1.283-1.416) | 9.60E-31 |  | 1.065 (1.005-1.129) | 1.304E-07 |

**Table S2. Univariate analysis and multivariate COX analysis of clinical prognostic parameters in TCGA dataset.**

| **Factors** | **Univariate analysis** | |  | **Multivariate analysis** | |
| --- | --- | --- | --- | --- | --- |
|  | **Hazard ratio (95% CI)** | ***P* value** |  | **Hazard ratio (95% CI)** | ***P* value** |
| Grade | 209.371 (29.360-1493.038) | 9.719 E-08 |  | 133.134 (18.545-955.758) | 1.153 E-06 |
| Age | 15.043 (6.077-37.280) | 4.584 E-08 |  | 3.554 (1.360-9.289) | 0.01 |
| Gender | 1.140 (0.737-1.764) | 0.56 |  | 0.622 (0.388-0.997) | 0.049 |
| IFI30 expression | 2.923 (2.341-3.648) | 2.568 E-21 |  | 1.898 (1.434-2.510) | 7.199 E-06 |

**2. Supplementary MATERIALS AND METHOD**

**2.1 Bioinformatics analyses**

IFI30 expression values, copy number variation data, and survival time data for glioma patients in this study were downloaded from online public databases, including TCGA (<http://cancergenome.nih.gov>) and CGGA (http://www.cgga.org.cn/), for bioinformatic analysis. IFI30 expression data for normal samples were downloaded from Genotype-Tissue Expression database (GTEx; <http://commonfund.nih.gov/GTEx/>) databases. All the data for each variable were normalized before statistical analyses. The GEPIA website (http://gepia.cancer-pku.cn/) was used to analyse the levels of IFI30 expression in different types of tumours and corresponding nontumor tissues.

**2.2 Ethics statement**

Glioma tissues, paracancerous (PA) tissues and normal brain tissues (NBT) were collected at the Second Affiliated Hospital of Soochow University. This study was performed in accordance with the principles of the Ethics Committee of Second Affiliated Hospital of Soochow University. The animal experiment complied with a protocol approved by the Institutional Animal Care and Use Committee of the Second Affiliated Hospital of Soochow University.

**2.3 Clinical samples**

Archived glioma tissues (WHO grades Ⅱ-Ⅳ, n=22) and PA (n=5) were gathered from glioma patients who underwent surgery and NBT (n=7) from severe traumatic brain injury patients who experienced partial resection of the normal brain as decompression treatment in the Department of Neurosurgery in the Second Affiliated Hospital of Soochow University from June 2017 to February 2020. One set of glioma tissue was fixed in formalin and paraffinized for immunohistochemical staining (IHC). The other set of glioma tissue for Western blotting was rapidly stored (stored at − 80 °C). None of the patients received any chemotherapy or radiotherapy before surgery. All patients signed informed consent forms.

**2.4 Cells, cell culture and reagents**

The human glioma cell lines (SNB19, T98G, GBM6, U87 and SF767) and Normal Human Astrocytes (NHA) were obtained from Research as a gift from Dr. Frank Furnari and Dr. Webster Cavennee (Ludwig Institute for Cancer Research, San Diego Branch). The human glioma stem-like cell lines (GSC464, GSC23, GSC206, GSC267, GSC62 and GSC11) and U87-EGFRvIII (U87-MG engineered with EGFRvIII by retrovirus transduction) were gifts from Dr. Li Ming (Department of Neurosurgery, University of Minnesota). Glioma cell lines and NHA cells were cultured in DMEM with 10% foetal bovine serum (FBS). Glioma stem-like cell lines were cultured in DMEM/F-12 medium containing glutamine, heparin (5 ng/ml), B27, FGF (20 ng/ml), and EGF (20 ng/ml). All of the cells were cultured at 37 °C and 5% CO2 without antibiotics. To monitor resistance to TMZ, which was purchased from Selleck and diluted in DMSO, glioma cells were treated with TMZ at various concentrations and times. This study determined the IC50 of temozolomide for U87-EGFRvIII and SNB19. We therefore used different concentrations of TMZ to select the TMZ-resistant (TR) of glioma cell lines (U87-EGFRvIII 500 μM and SNB19 50 μM) for 3 months. During this period, temozolo-mide-containing media were replaced at 3-day intervals. The corresponding methods were mainly based on the previous study of Zhou J et al[1] and with minor adjustment in this study. The Akt activator SC79, Akt inhibitor MK-2206 and EGFR inhibitor AG-1478 were purchased from Selleck and diluted in DMSO. For the inhibitor and activator experiments, MK-2206 (10 mM) was added to the medium for 18-24 h; SC79 (10 µ g/mL) was added to the medium for 1-2 h; AG-1478 (10 mM) was added to the medium for 1h.

**2.5 Plasmids and reagents**

The human IFI30 and Slug genes fused with 3 × Flag at the C-terminus were cloned into the pcDNA3.1 vector. All plasmids and blank genes with 3 × Flag at the C-terminus in the pcDNA3.1 vector were synthesized by GENEWIZ Co. (Suzhou, China). Short hairpin RNA (shRNA) targeting IFI30 and an NC shRNA were purchased from GenePharma Co. (Suzhou, China). The IFI30 shRNA-1# sequence was 5′-CCAGACACTATCATGGAGTGT-3′; the IFI30 shRNA-2# sequence was 5′-CCCTACGGAAACGCACAGGAA-3′; The NC shRNA sequence was 5′-TTCTCCGAACGTGTCACGT-3′. The Slug siRNA sequence was 5′-GGACCACAGUGGCUCAGAATT-3′ and NC siRNA sequence was 5′-UUCUCCGAACGUGUCACGU-3′ was purchased from GenePharma Co. (Suzhou, China).

**2.6 Virus production and infection**

The 293FT cells were transfected with pLKO.1-shGFP or pLKO.1-shIFI30 and the helper vectors pCMVDR8.91 and pMD.G-VSV-G using Lipofectamine 3000 reagent. The culture medium was changed with fresh DMEM/10% FBS after 24 h. The various supernatants were collected every 24 h for 3 days and filtered with a 0.45 μm nitrocellulose filter. Glioma cells were infected with lentivirus supernatant for 48 h and selected with puromycin (4 μg/ml) for 10 days to obtain stable pooled clones. The stable pooled clones were verified by immunoblotting.

**2.7 Gene transfection**

When the cells plated in a six-well plate reached the appropriate density, plasmids and siRNA were transiently transfected with Lipofectamine 3000 (Invitrogen) and Lipofectamine RNAiMAX (Invitrogen) according to the manufacturer’s instructions. Forty-eight hours after transfection, the cells were used for further experiments.

**2.8 RNA isolation and RT-PCR**

Total RNA was extracted using TRIzol regent (Invitrogen, USA). We used the SuperScript First-Strand cDNA System (Thermo Fisher Scientific, USA) to prepared for cDNA, and real-time PCR was performed by using SYBR Green (Vazyme, China) according to the manufacturer’s protocol. GAPDH was used for normalization, and the comparative Ct method (ΔΔCt) was used to evaluate mRNA expression. The specific primer pairs used were presented in Table 1.

**Table 1. List of primers used in this study.**

| Primers | Sequence (5'-3') |
| --- | --- |
| Primers for qRT-PCR |  |
| IFI30 | Forward: 5′-GACCGAGAAACTGAGCTCCC C-3′ |
|  | Reverse: 5′-TGGCATCGAACATCTGCTGG-3′ |
| EGFR | Forward: 5′-AAGGAAATCCTCGATGAAGCCT-3′ |
|  | Reverse: 5′-TGTCTTTGTTCCCGGACATA-3′ |
| Slug | Forward: 5′-ACTGGACACACATACAGTGATT-3′ |
|  | Reverse: 5′-ACTCACTCGCCCCAAAGAT-3′ |
| β-Actin | Forward: 5′-CATGTACGTTGCTATCCAGC-3′ |
|  | Reverse: 5′-CTCCTTAATGTCACGCACGAT-3′ |
| Vimentin | Forward: 5′-GAGAACTTTGCCGTTGAAGC-3′ |
|  | Reverse: 5'-TCCAGCAGCTTCCTGTAGGT-3' |
| E-cadherin | Forward: 5'-GAACGCATTGCCACATAC-3' |
|  | Reverse: 5'-ACCTTCCATGACAGACCC-3' |
| Nanog | Forward: 5'-GACAGTTACGCGCACATGAA-3' |
|  | Reverse: 5'-ACACAGCTGGGTGGAAGAGA-3' |
| Sox2 | Forward: 5'-GCGGAAAACCAAGACGCTC-3' |
|  | Reverse: 5'-TCAGCGCGTAACTGTCC-3' |
| Oct-4 | Forward: 5'-GACAACAATGAAAATCTTCAG GAG-3' |
|  | Reverse: 5'-TTCTGGCGCCGGTTACAGAAC CA-3' |

**2.9 Western blotting**

All protein samples from tissues and cells were lysed in a modified RIPA buffer (Beyotime Biotechnology, China) mixed with PMSF (100×) and protease inhibitor Cocktail (100×, Cell Signaling Technology, USA) and PhosSTOPEASYpack (Roche, Switzerland) On ice for about 30 min, then centrifuged at 12,000 rpm for 15 min. The concentration of the sample was quantitatively determined by BCA protein assay. The lysate was mixed with loading buffer after heating at 100 °C for 5-10 mins. Equal amounts of proteins were separated by SDS‒PAGE and transferred to nitrocellulose membranes. Then, the membranes were blocked in 5% skim milk for at least 1 h. The membranes were incubated with primary antibodies at 4 °C overnight and then with secondary antibodies in a shaded environment at room temperature for 1 h. An enhanced chemiluminescence system (Millipore, USA) was used to measure the protein expression value. The relative quantity of proteins was analysed by ImageJ software. The primary antibodies used are presented in Table 2. Western blot analysis was repeated three times.

**Table 2. List of antibodies used in this study.**

| **Antigen** | **Cat. No** | **Company** | **Dilution** |
| --- | --- | --- | --- |
| **Antibodies for WB** |  |  |  |
| IFI30 | sc-393507 | Santa Cruz | 1:500 |
| Slug | 12129-1-AP | Proteintech | 1:1000 |
| Vimentin | 10366-1-AP | Proteintech | 1:2000 |
| E-Cadherin | 20874-1-AP | Proteintech | 1:2000 |
| Akt | 9272 | CST | 1:1000 |
| p-Akt (Ser473) | 4060 | CST | 1:2000 |
| EGFR | sc-373746 | Santa Cruz | 1:1000 |
| p-EGFR (Tyr1068) | 3777 | CST | 1:1000 |
| Cleaved-PARP | 9532S | CST | 1:1000 |
| Oct-4 | 2750 | CST | 1:1000 |
| Sox2 | 3579 | CST | 1:1000 |
| Nanog | 14295-1-AP | Proteintech | 1:1000 |
| α-Tubulin | 11224-1-AP | Proteintech | 1:5000 |
| β-actin | A5441 | Sigma | 1:5000 |
| **Antibodies for IHC** |  |  |  |
| IFI30 | sc-393507 | Santa Cruz | 1:100 |
| Ki67 | GB111499 | Servicebio | 1:800 |
| p-Akt (Ser473) | 4060 | CST | 1:100 |
| EGFR | sc-373746 | Santa Cruz | 1:100 |
| Caspase 3 | 9661S | CST | 1:400 |
| **Antibodies for IF** |  |  |  |
| IFI30 | sc-393507 | Santa Cruz | 1:200 |
| Slug | 12129-1-AP | Proteintech | 1:1000 |
| Vimentin | 10366-1-AP | Proteintech | 1:200 |
| E-Cadherin | 20874-1-AP | Proteintech | 1:200 |
| CD44 | 3570 | CST | 1/400 |
| ESA | ab96507 | Abcam | 1/100 |
| **Antibodies for FC** |  |  |  |
| ESA | ab307422 | Abcam | 1:50 |
| Anti-Human/Mouse CD44, PE | F1104402 | MULTI SCIENCES | 5 μL |

**2.10 Immunohistochemical (IHC) staining and haematoxylin–eosin (H-E) staining**

Formalin-fixed tumour samples and tissues from xenograft mice were embedded in paraffin and sliced into 5 μm thick sections. Next, the sections were immunostained with the indicated primary antibodies at 4 °C overnight, followed by incubation with the secondary antibody for 30 min at 37 °C. Then, the tissue slides were visualized using diaminobenzidine (DAB, Sigma Aldrich, USA) for 15 min. After washing, the sections were counterstained with haematoxylin, dehydrated and cover slipped following the manufacturer’s protocols. The primary antibodies used are presented in Supplementary Table 2. For HE staining, the paraffin-embedded brains of xenograft mice were sequentially deparaffinized, dehydrated, stained with haematoxylin, differentiated by the addition of hydrochloric ethanol, returned to blue with ammonia water, and stained with eosin. Then, the sections were dehydrated with gradient alcohol, cleared with xylene, and sealed with neutral resin. The final intensity of each staining was quantified using ImageJ software. The positive staining rate was scored as follows: High positive (3), Positive (2), Low Positive (1) and Negative (0).

**2.11 Limiting dilution analysis**

GSC267 or GSC464 cell spheres were trypsinized into individual cells with Accutase trypsin (Invitrogen, USA) and were counted and cultured in 96-well plates in ten plates containing 100 µL of GSC medium at different densities (20, 100, 200, 500, 1000 and 1500 cells/well). The formation of tumour spheres was determined in each well after 7-10 days, and GSC frequency was calculated by extreme limiting dilution analysis (<http://bioinf.wehi.edu.au/software/elda/>).

**2.12 Xenograft tumor assay**

Five-week-old female athymic nude mice (BALB/c) (SLAC Laboratory Animal Company, China) (5 mice for each group) were divided randomly before injection. For the subcutaneous model, 2.0×10^6^ GSC267-OE-IFI30/GSC464-shNC and GSC267-NC/GSC464-shIFI30 stable cells resuspended in PBS and Matrigel were subcutaneously injected into the mice. The mice were euthanized with ketamine and xylazine to collect the tumours. The excised tumours were then photographed. The tumour volume was calculated by the formula V = (a×b2)/2 (a and b represent the long and short diameters of the tumour, respectively). For the intracranial glioblastoma model, 5×10^5^ GSC267-OE-IFI30/GSC464-shNC and GSC267-NC/GSC464-shIFI30 cells were suspended in 10 μl PBS and injected into the right brain hemisphere of the mice with a stereotaxic system (randomly assigned into 6 mice/group). Mice were observed daily for death or neurological symptoms and then sacrificed if they developed neurological symptoms. The entire brain was then collected, fixed with 4% PFA, embedded in paraffin, and sectioned coronally from anterior to posterior. For survival analysis assays, mice (6 mice for each group) were injected intracranially with the above procedure. The dying mice were sacrificed under deep anaesthesia, and the remaining mice were sacrificed 90 days after injection of the same cells. For in vivo TMZ treatment, mice (6 mice for each group) were treated with TMZ by intragastric administration for 5 days (60 mg/kg/day) at 7 days post implantation. For in vivo EGFR inhibitor (AG-1478) treatment, mice (5 mice for each group) were were treated i.p. three times per week for 2 weeks with 800 μg of AG1478 in subcutaneous models of GSC267-OE-IFI30 cells to determine its efficacy in IFI30-mediated the EMT-like process.

**2.13 Immunofluorescence microscopy**

The cells were fixed with 4% formaldehyde for 15 min at 37 °C and permeabilized with 0.1% Triton X-100 (made in PBS, Thermo Fisher Scientific, USA) for 10 min at 4 °C. Then, the slides were washed with PBS 3 times and 5% goat serum (Sigma, Japan) blocked at RT for 1 h. The cells were then incubated with the indicated primary antibodies at 4 °C overnight, followed by incubation with fluorescence-conjugated secondary antibodies at room temperature for 1 h in the dark. Finally, the cell nuclei were stained with DAPI. The images were observed using a Zeiss confocal microscope (Carl Zeiss, Germany). The primary and secondary antibodies are shown in Table 2.

**2.14 Cell Counting Kit-8 (CCK-8) assay and** **clone formation**

For the CCK-8 assay, 1000 cells were resuspended in 100 μl DMEM supplemented with 10% FBS and then added to a 96-well plate with or without TMZ and cultured for the indicated time. Cell proliferation was investigated using CCK-8 (Selleck Chemicals LLC, USA) according to the manufacturer’s instructions. The plate was read on a microplate reader (TECAN, Switzerland) at 450 nm wavelength. A total of 400-700 glioma cells were counted and seeded in 6-well plates. Cells were cultured with complete medium and then with or without TMZ (50 or 20 μM/12 h). The medium was replaced after TMZ treatment, and the cells were washed with PBS. Cells were cultured in complete medium for approximately 10 days. Colonies were stained with 0.2% crystal violet (Sigma-Aldrich, USA).

**2.15 Migration and transwell** **assay**

A migration test also analyzed the cell migration ability. A total of 2×104 cells were seeded in the upper chamber in serum-free medium. Then, 500 μL of complete medium was added to the lower chamber. After 24 h of incubation, the cells were fixed with 4% formaldehyde at room temperature for 10 min. After staining with 0.2% crystal violet, the number of invasive cells was counted. Analysis of cancer cell invasion ability was performed via a transwell chamber assay. In the invasion experiment, Matrigel (DMEM 1:8 dilution, Corning, USA) was precoated in a transwell chamber (Corning, USA) and soaked at 37 °C for 2 h before the experiment. The next steps were performed as in the migration test method.

**2.16 Flow cytometric analysis**

Cells were seeded in a 6-well plate and treated with temozolomide (50-400 μM) for 48-72 h. An Annexin V-PE/7- ADD kit (Mulit Sciences, China) was used to measure the apoptosis of glioma cells. All operations were carried out according to the manufacturer’s instructions. In brief, cells were harvested and washed three times with PBS. Then, the cells were stained with Annexin V-PE/7- ADD for 10 min in the dark and detected with a CytoFLEX flow cytometer (Beckman, USA). Early apoptosis and late apoptosis rates were summed, and the total apoptosis rate was calculated. Gliospheres were incubated with Accutase and repeatedly pipetted with a pipette to disperse the spheres into a single state, and washed twice with cold PBS. The cells were centrifuged and resuspended in binding buffer, The cells were stained with ESA antibody on ice for 30 min and then CD44-PE antibody and anti-IgG APC (**A-865**) (invitrogen) were added and incubated in the dark at room temperature for 15 min. The samples were then analyzed by a CytoFLEX flow cytometer (Beckman, USA).

**2.17 Wound healing assay**

The wound healing assay was used to prove the cell migration ability. Same numbers cells were seeded in the 6-well plates. The wound was created by the 200 μl sterile pipette tips when the cell reached 80%-90% confluence. After scratching, the detached cells were washed gently by PBS and cultured with serum-free medium. Cell migration distance was observed at four different spot and pictured at 0, 12, 24, 48h at the same place. Cell migration index =migrated distance (at indicated time)/original scratching distance (at 0 h).

**2.18 Statistical analysis**

The data are presented as the mean ± standard deviation (SD) from at least three independent experiments. The normality of the data distribution was analyzed by the Shapiro–Wilk test. Two-tailed Student’s t test was used to analyze the differences in the results between the two groups. Comparisons among three or more groups were performed by using one-way analysis of variance. A survival curve was prepared by Kaplan–Meier analysis. The statistical analyses were performed with SPSS 24.0 (SPSS Inc., Chicago) and GraphPad Prism version 8 (GraphPad Software, USA). A *p* value < 0.05 was considered to indicate statistically significant results. **p*<0.05, ***p*<0.01, ****p*<0.001, *****p*<0.0001

**REFERENCES**

1. Zhou J, Xu N, Liu B, Wang C, He Z, Lenahan C, et al. lncRNA XLOC013218 promotes cell proliferation and TMZ resistance by targeting the PIK3R2-mediated PI3K/AKT pathway in glioma. Cancer science. 2022;113(8):2681-92.


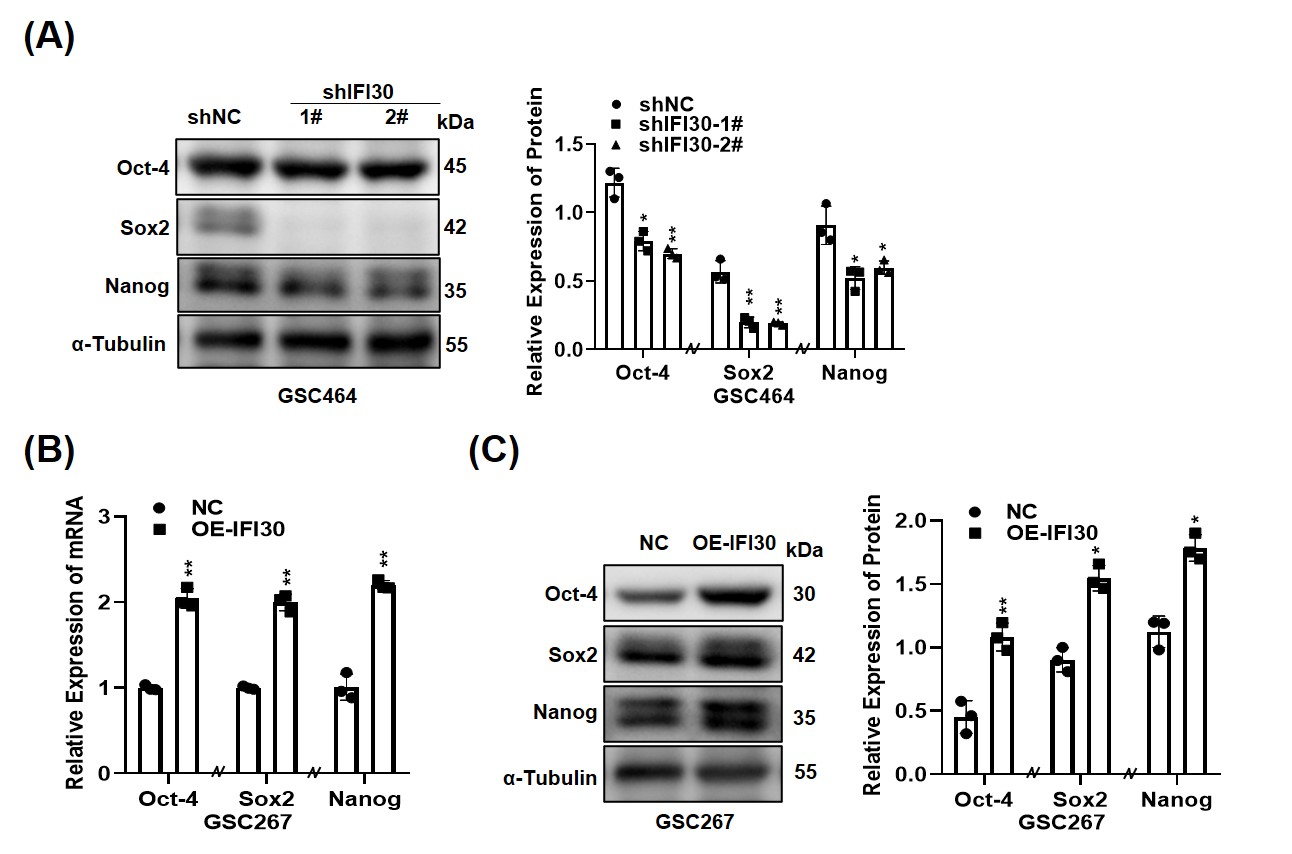


**Figure S1** IFI30 can increase the stemness of glioma. (A) The protein levels of Oct-4/Sox2/Nanog in the control and IFI30 knockdown GSC464 cells. (B) The mRNA and (C) protein levels of Oct-4/Sox2/Nanog in GSC267 cells with or without IFI30 overexpression.

**
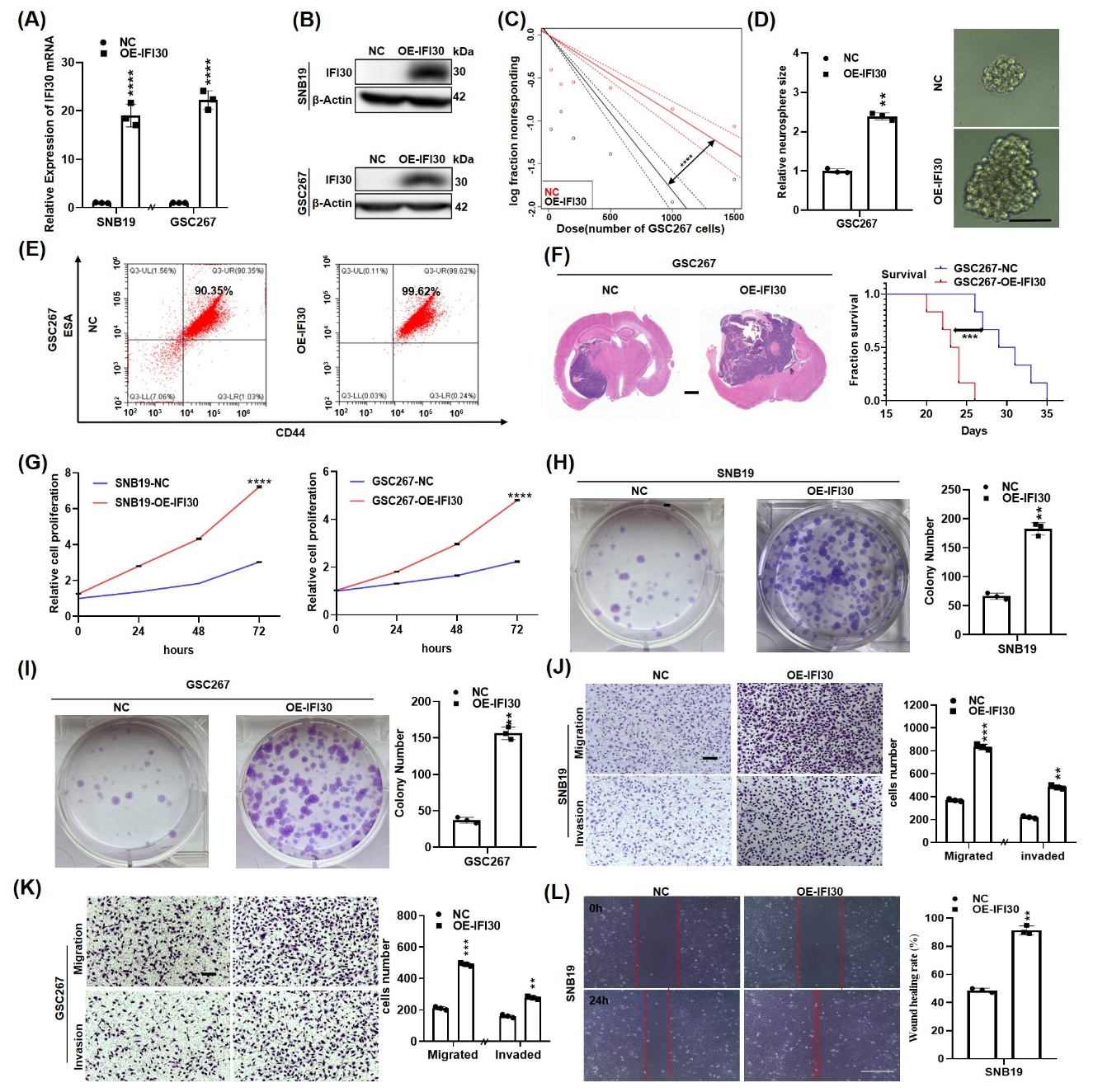
**

**Figure S1** IFI30 over-expression accelerated the proliferation, migration and invasion of glioma cells. (A) The mRNA and (B) protein levels of IFI30 in SNB19 and GSC267 cells with or without IFI30 overexpression. (C) A limiting dilution assay was performed in GSC267 cells with or without IFI30 overexpression. (D) Representative images of sphere formation capability of GSC267 cells with or without IFI30 overexpression. Bar = 100 μm. (E) Expression of CD44 and ESA in the control and IFI30 overexpression GSC267 cells according to Flow cytometry analyses. (F) Representative H&E staining of brain sections on day 20 after intracranial inoculation of GSC267-NC or GSC267-OE-IFI30 in nude mice. 5×10^5^ cells/mouse, 6 mice/group. Bars=100 μm. Kaplan–Meier survival curve of mice. Cell proliferation was measured using (G) CCK-8 and (H and I) colony formation assays. The migration and invasion capabilities were examined by Transwell assays in SNB19 (J) and GSC267 (K) cells with or without IFI30 overexpression. Bar=200 µm. The number of migrated and invasive cells was quantified. (L) Wound healing analysis was used to determine the migration of SNB19-NC/OE-IFI30 cells. Bar=400 µm. The wound closure percentage was calculated by ImageJ software. All panels show the mean ± SD of three independent experiments; ***p*<0.01, ****p*<0.001, *****p*<0.0001.

**
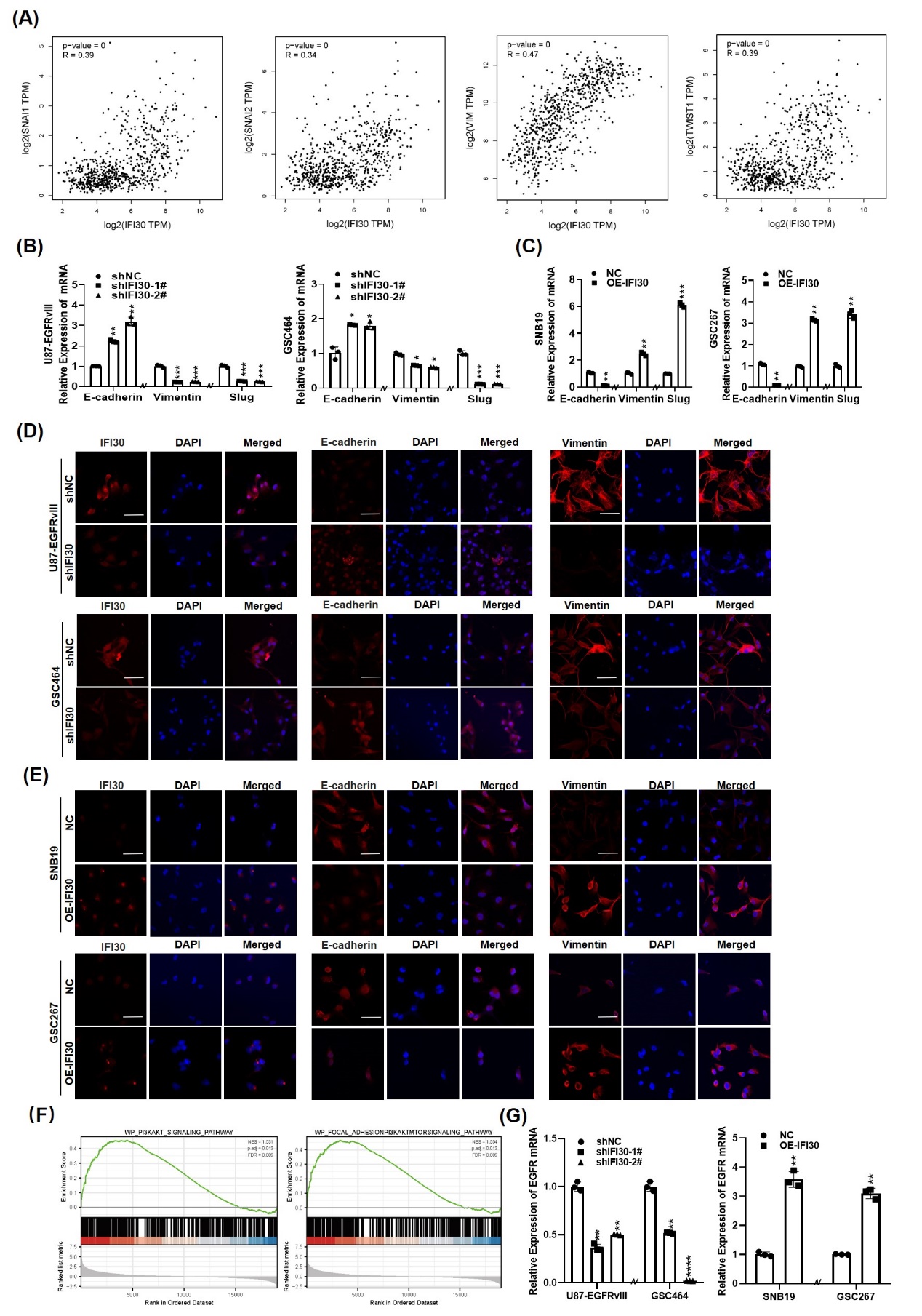
**

**Figure** **S3** IFI30 promoted the EMT-like process and activated the EGFR/AKT/GSK3β/β-catenin signalling pathway in glioma cells in vitro and in vivo. (A) Correlation between IFI30 and EMT-related markers in TCGA. Pearson’s test was used for correlation analysis. The mRNA levels of Slug, E-cadherin and Vimentin after (B) knockdown or (C) overexpression of IFI30 in glioma cells. IFI30, E-cadherin and Vimentin immunofluorescence staining images of (D) IFI30-silenced or (E) IFI30-overexpressing cells were captured by confocal microscopy. Bar=100 μm. (F) The mRNA levels of EGFR in IFI30-knockdown or IFI30-overexpressing glioma cells. (G) GSEA results for Akt-related genes in tissues with high IFI30 expression from TCGA. All panels show the mean ± SD of three independent experiments; **p*<0.05, ***p*<0.01, ****p*<0.001, *****p*<0.0001.


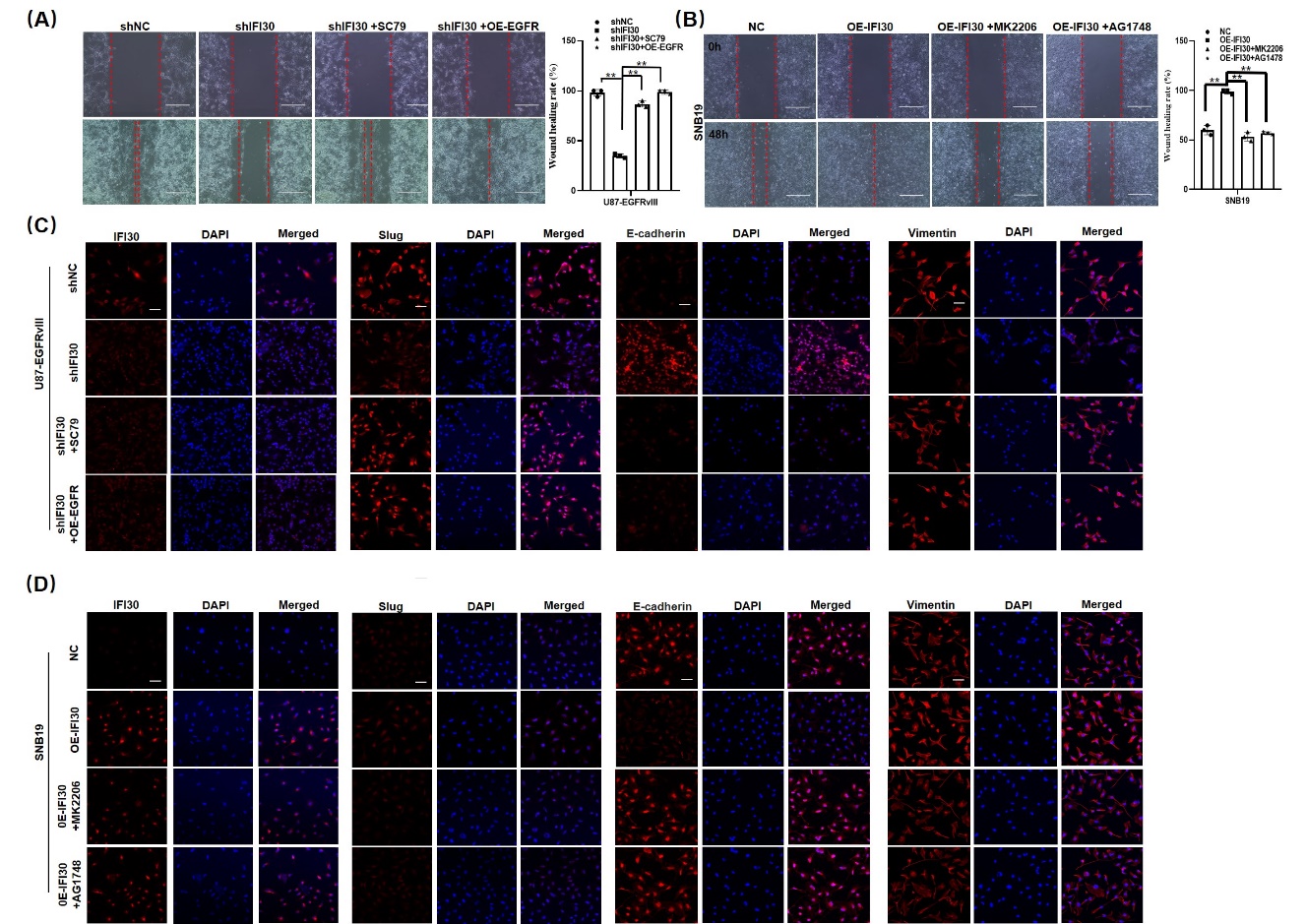


**Figure S4** The EGFR/Akt signalling pathway is essential for the IFI30-mediated EMT-like process. Wound healing analysis was used to detect the migration abilities of shIFI30 cells treated with (A) EGFR plasmid and SC79 or (B) OE-IFI30 cells treated with AG-1478 and MK-2206. Bar=400 μm. The wound closure percentage was calculated by ImageJ software. Immunofluorescence staining was used to detect the expression of IFI30, vimentin, Slug and E-cadherin in shIFI30 cells treated with (C) EGFR plasmid and SC79 or OE-IFI30 cells treated with (D) AG-1478 and MK-2206. Bar=100 µm. All panels show the mean ± SD of three independent experiments; ***p*<0.01.**
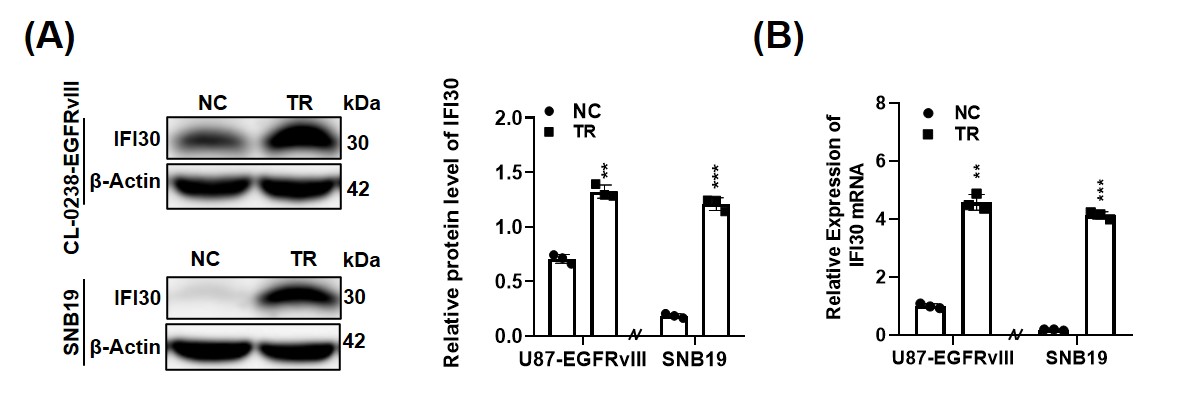
**

**Figure S5.** IFI30 was involved in the TMZ resistance process. (A) Western blotting was used to detect the expression of IFI30 in U87-EGFRvIII TR and SNB19 TR cell lines. Quantification of the protein expression was performed with ImageJ software; β-Actin was used as a loading control. (B) The mRNA levels of IFI30 in I U87-EGFRvIII TR and SNB19 TR cell lines. All panels show the mean ± SD of three independent experiments; ***p*<0.01, ****p*<0.001.

**
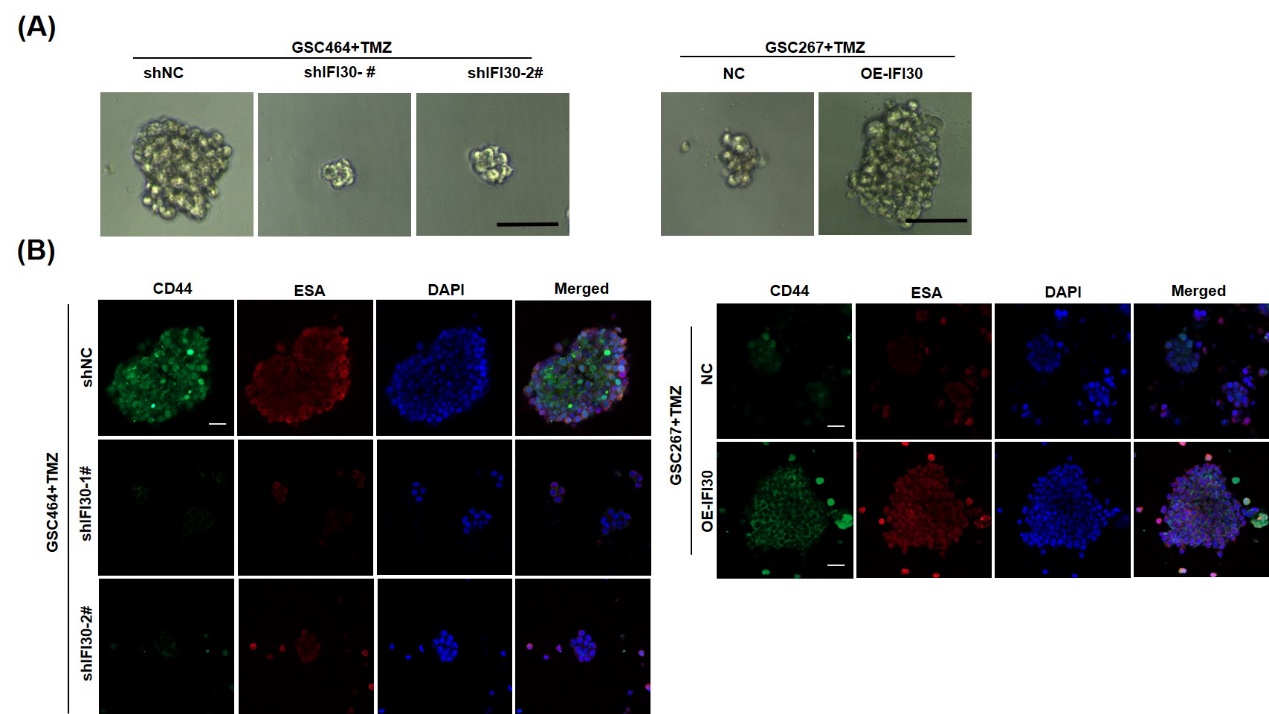
**

**Figure S6.** IFI30 can increase the stemness and TMZ resistance of glioma. (A) Sphere assay and (B) CD44/ESA staining in GSC464 and GSC267 cells in both silenced and overexpressed settings treated with different doses of TMZ.
