# Supplementary figures and images for: Interferon‐γ inducible protein 30 promotes the epithelial–mesenchymal transition‐like phenotype and chemoresistance by activating EGFR/AKT/GSK3β/β‐catenin pathway in glioma
Source: CNS Neurosci Ther. 2023 Jul 5;29(12):4124–38. doi: 10.1111/cns.14334 (PMC10651985; doi:10.1111/cns.14334)

**Original Western blotting images**

**
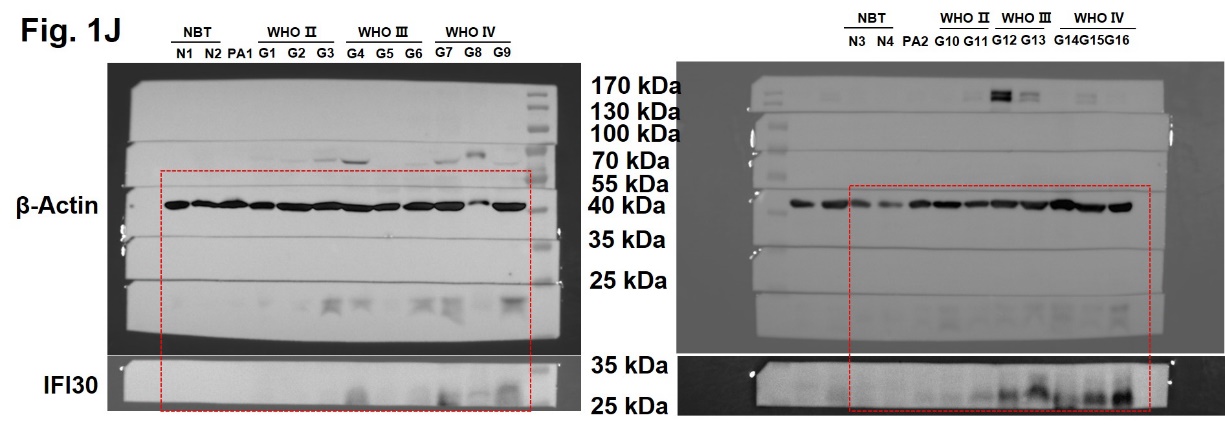
**

**
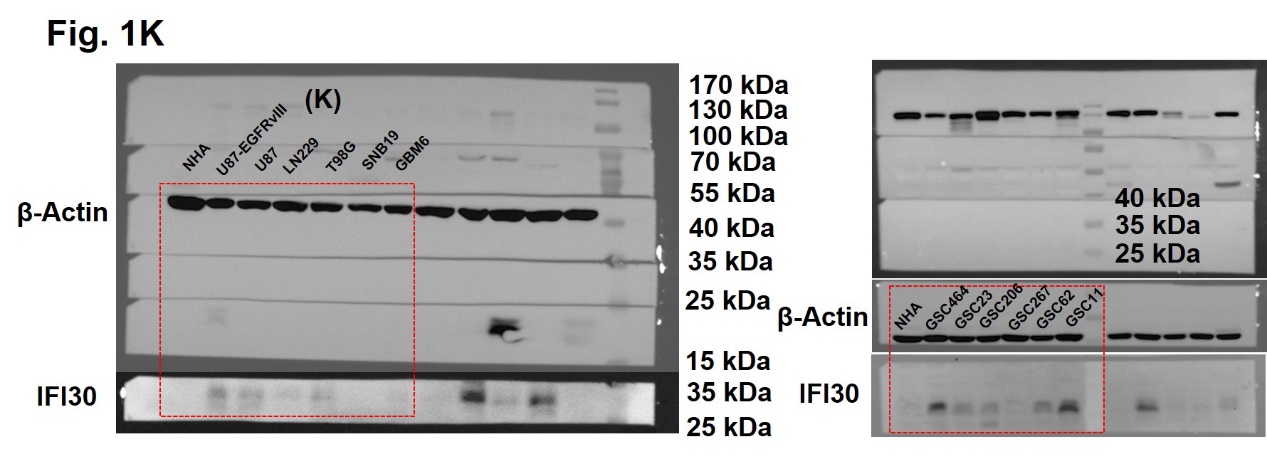
**

**
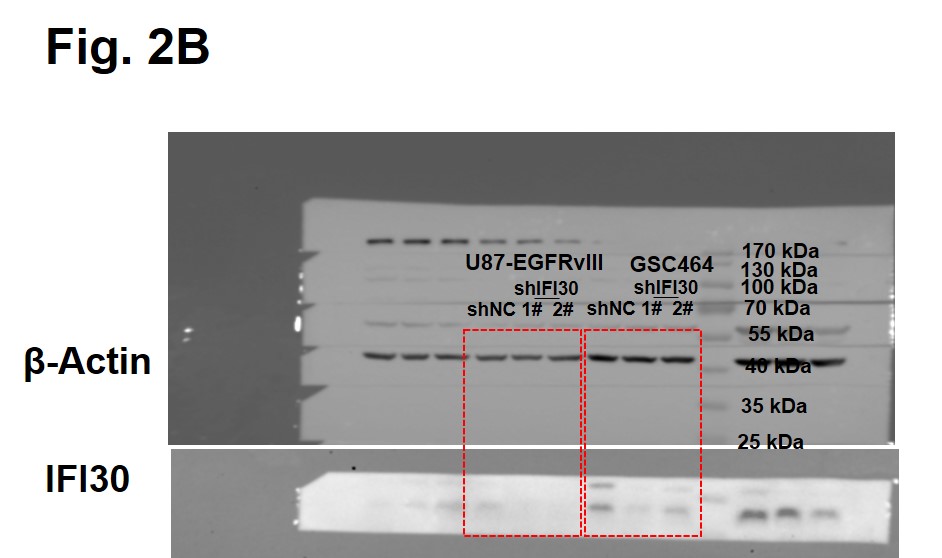
**

**
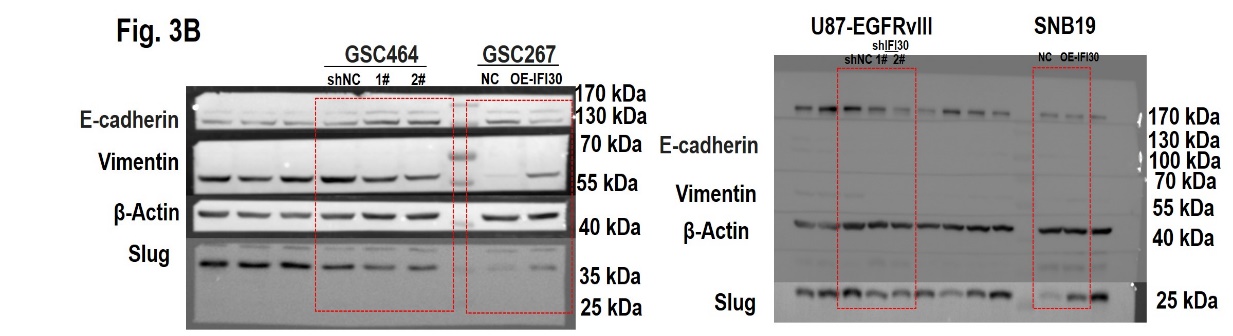
**

**
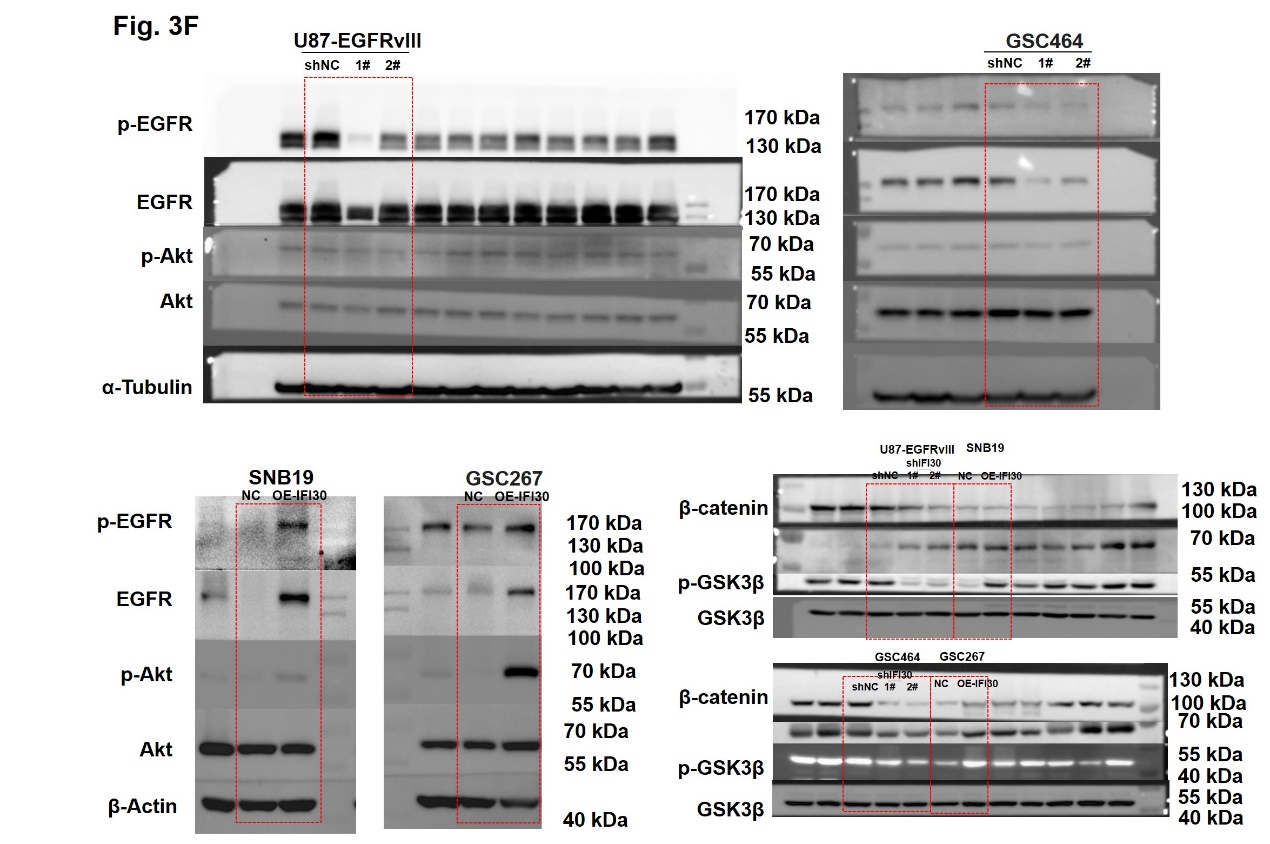
**

**
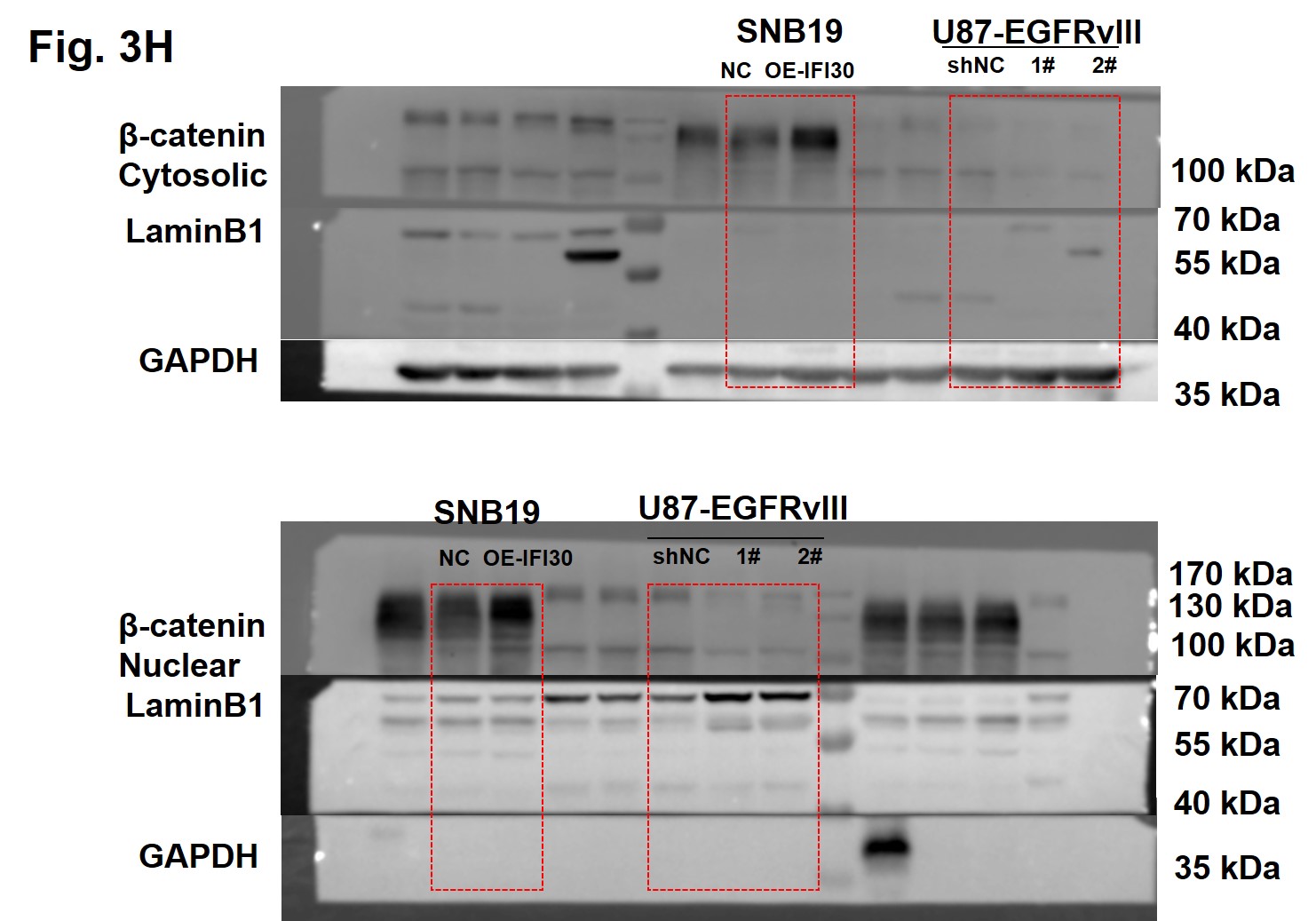
**

**
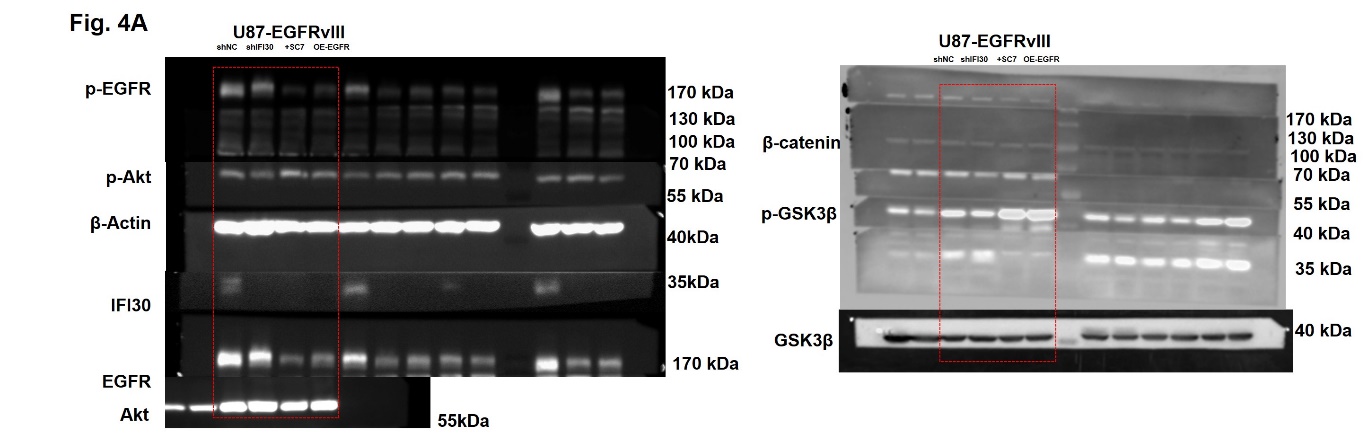
**

**
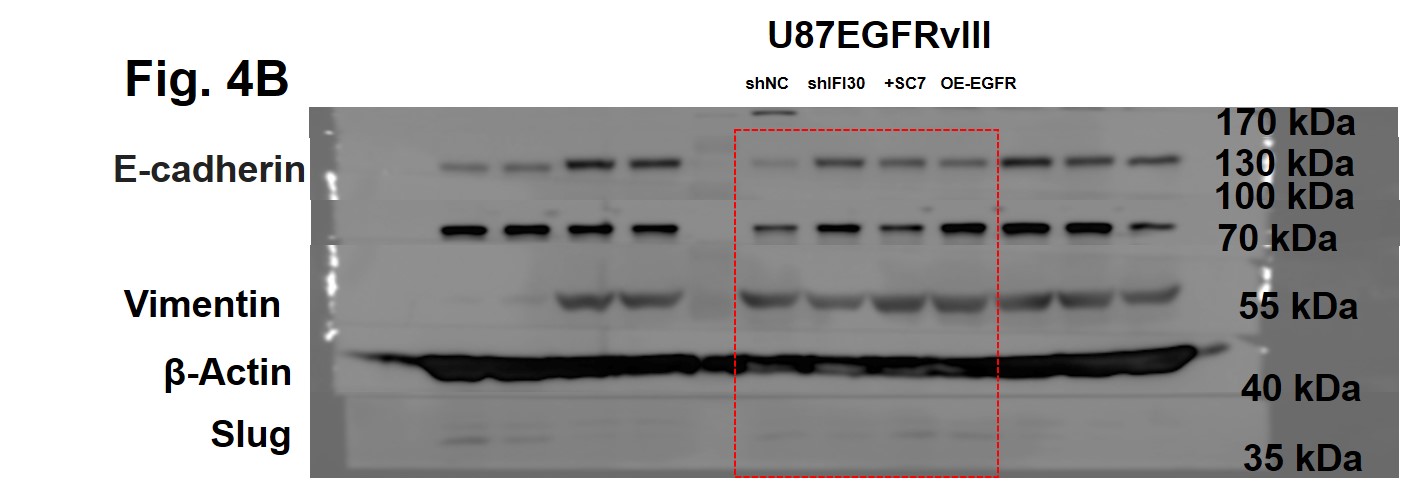
**

**
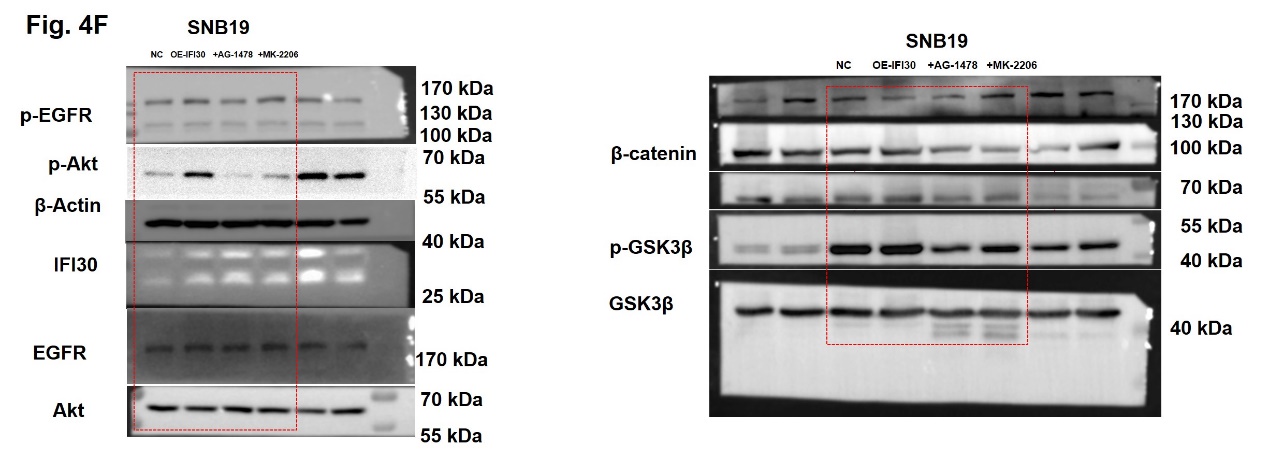
**

**
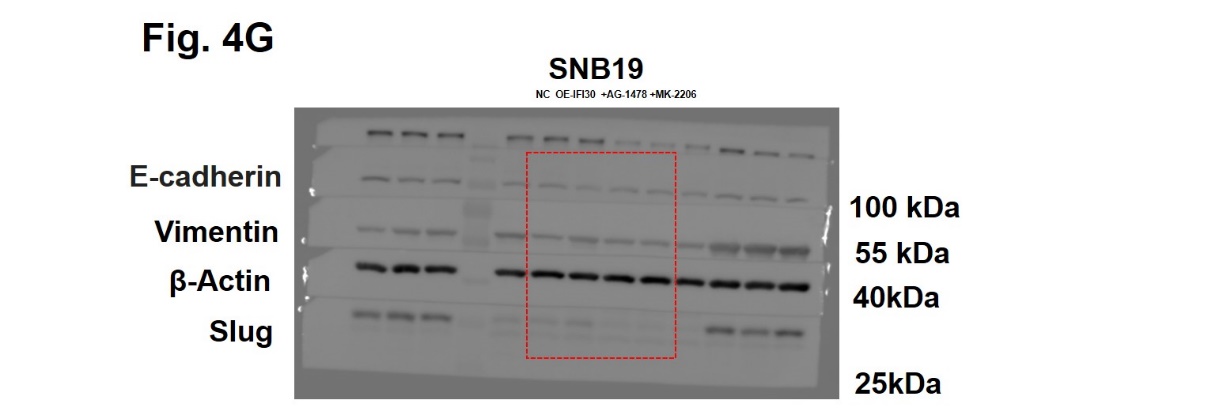
**

**
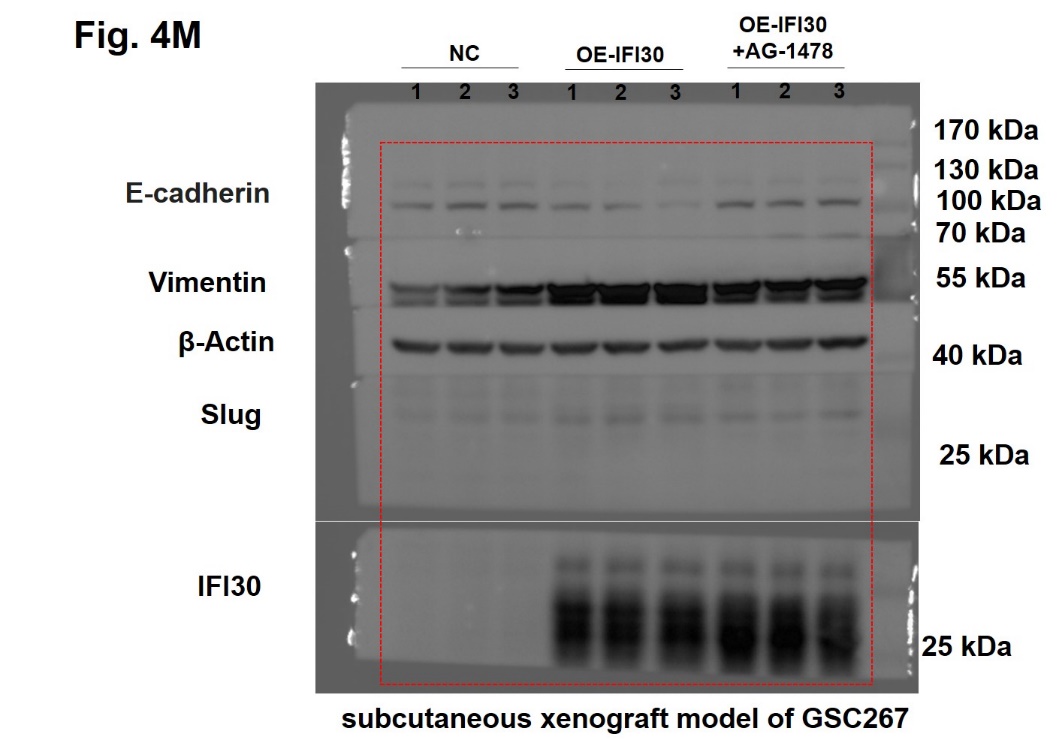
**


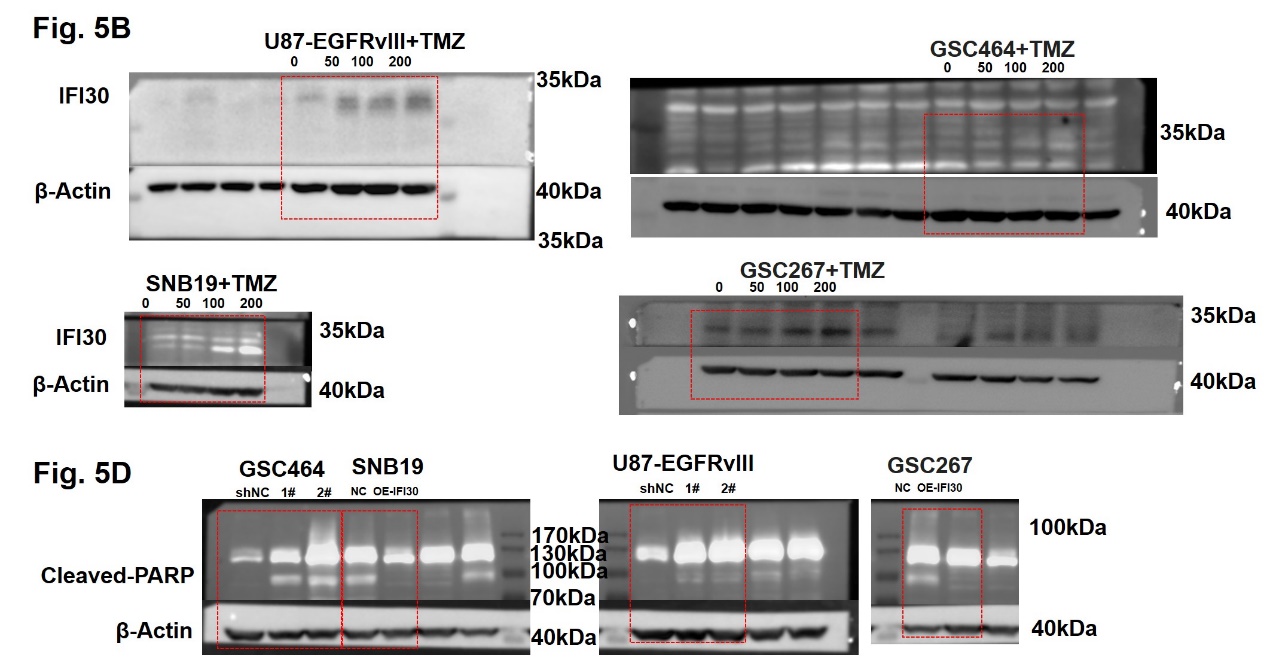


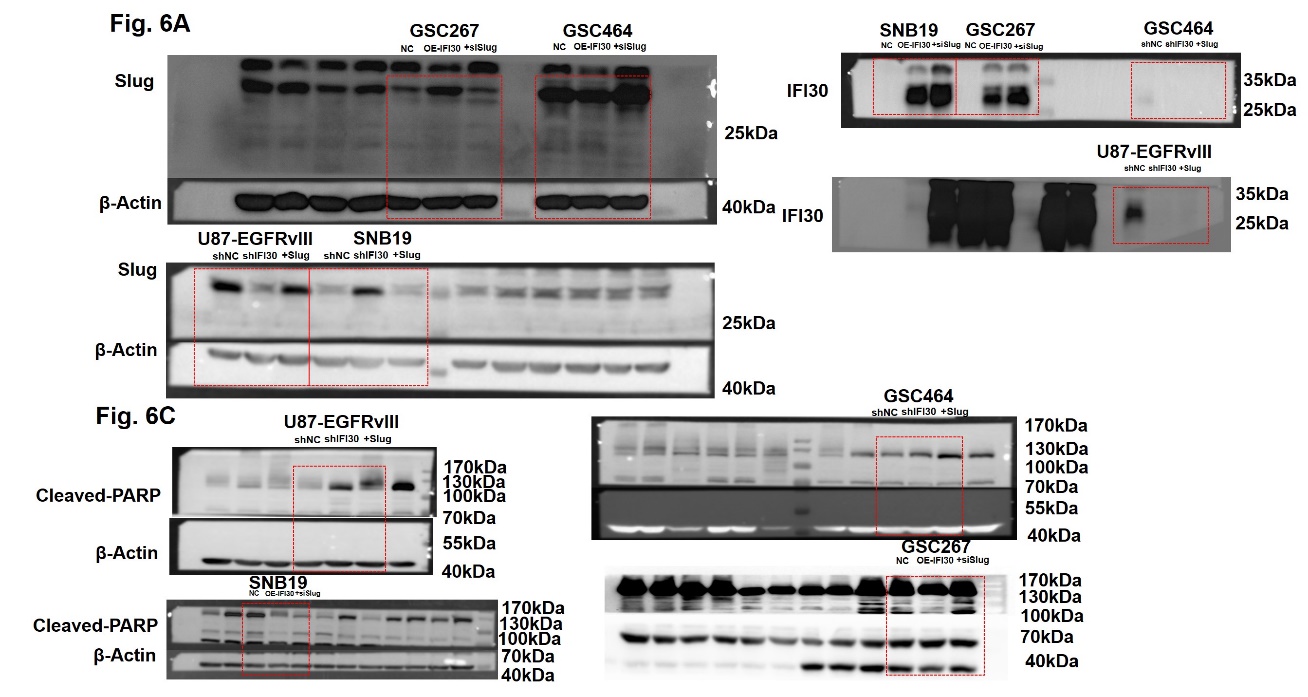

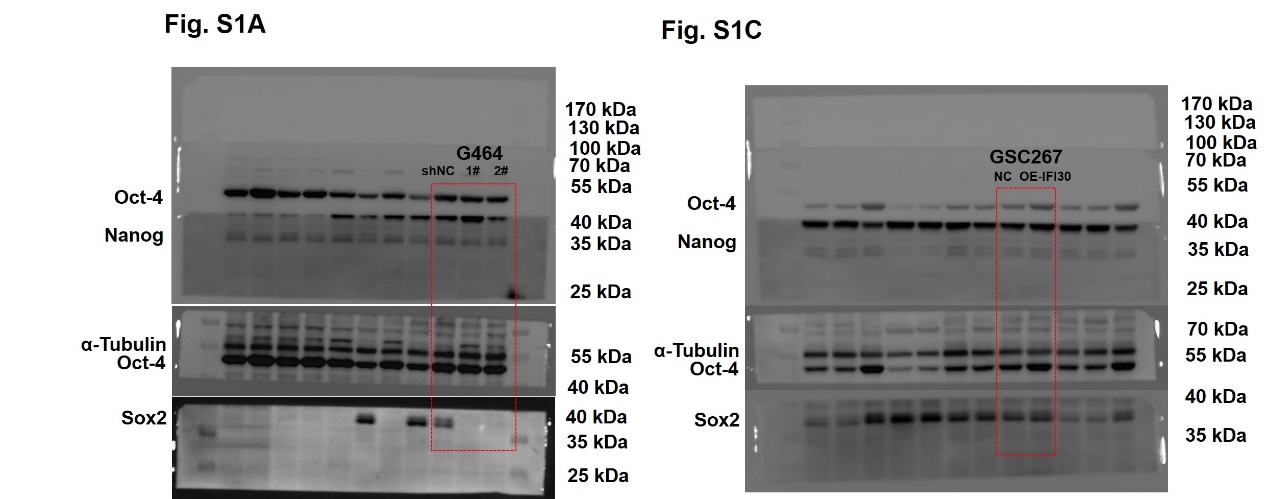


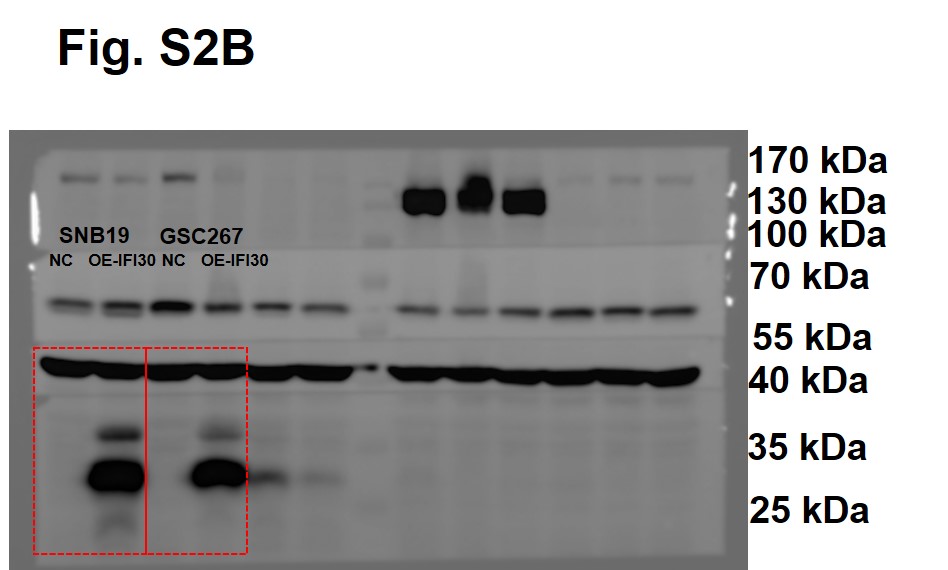


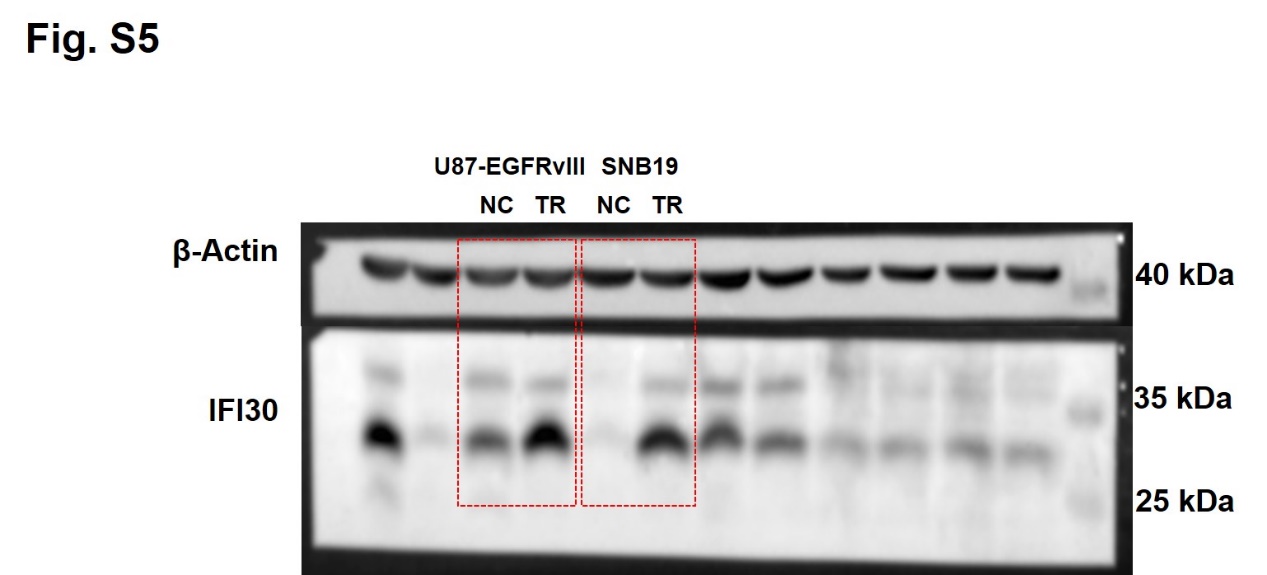

Supplement: Supplementary file 2 — Data S2. [file CNS-29-4124-s002.docx]
